# Ulcerative colitis: molecular insights and intervention therapy

**DOI:** 10.1186/s43556-024-00207-w

**Published:** 2024-10-10

**Authors:** Yuqing Liang, Yang Li, Chehao Lee, Ziwei Yu, Chongli Chen, Chao Liang

**Affiliations:** 1 School of Basic Medical Sciences, Chengdu University of Traditional Chinese Medicine, Chengdu, 611137 China; 2 Department of Geriatrics, Hospital of Chengdu University of Traditional Chinese Medicine, Chengdu, 610072 China; 3 Department of Respiratory, Sichuan Integrative Medicine Hospital, Chengdu, 610042 China; 4 Department of Traditional Chinese Medicine, Key Laboratory of Birth Defects and Related Diseases of Women and Children of Ministry of Education, West China Second University Hospital, Sichuan University, Chengdu, 610041 China; 5 State Key Laboratory of Southwestern Chinese Medicine Resources, College of Pharmacy, Chengdu University of Traditional Chinese Medicine, Chengdu, 611137 China

**Keywords:** Ulcerative colitis (UC), Genetic predisposition, Immune dysregulation, Alteration of intestinal flora, Biologic therapy, Herbal treatment

## Abstract

Ulcerative colitis (UC) is a chronic inflammatory bowel disease characterized by abdominal pain, diarrhea, rectal bleeding, and weight loss. The pathogenesis and treatment of UC remain key areas of research interest. Various factors, including genetic predisposition, immune dysregulation, and alterations in the gut microbiota, are believed to contribute to the pathogenesis of UC. Current treatments for UC include 5-aminosalicylic acids, corticosteroids, immunosuppressants, and biologics. However, study reported that the one-year clinical remission rate is only around 40%. It is necessary to prompt the exploration of new treatment modalities. Biologic therapies, such as anti-TNF-α monoclonal antibody and JAK inhibitor, primarily consist of small molecules targeting specific pathways, effectively inducing and maintaining remission. Given the significant role of the gut microbiota, research into intestinal microecologics, such as probiotics and prebiotics, and fecal microbiota transplantation (FMT) shows promising potential in UC treatment. Additionally, medicinal herbs, such as chili pepper and turmeric, used in complementary therapy have shown promising results in UC management. This article reviews recent findings on the mechanisms of UC, including genetic susceptibility, immune cell dynamics and cytokine regulation, and gut microbiota alterations. It also discusses current applications of biologic therapy, herbal therapy, microecologics, and FMT, along with their prospects and challenges.

**Supplementary Information:**

The online version contains supplementary material available at 10.1186/s43556-024-00207-w.

## Introduction

Ulcerative colitis (UC) is a chronic inflammatory bowel disease (IBD) that manifests with inflammation and ulcers in the colonic lining, resulting in a spectrum of symptoms including abdominal pain, diarrhea, rectal bleeding, tenesmus (a sense of pressure), and weight loss [1, 2]. Disease activity in UC is typically characterized by a pattern of relapses and remission, and ​remission is marked by alternating phases of active symptoms and clinical quiescence [3, 4]. Furthermore, UC can, in certain instances, give rise to complications such as colon perforation, bleeding, or an elevated risk of colon cancer [5–8].

In 2023, the global prevalence of UC was estimated at approximately 5 million cases, imposing substantial health and economic burdens [9]. The etiology of UC is multifactorial, involving complex interactions among genetic predisposition, immune dysregulation, environmental factors, and gut microbiota alterations [10, 11]. All these factors play a crucial role in UC, involved in immune and inflammatory responses, epithelial barrier function, and microbial interactions of the disease. Present recommended medications for the treatment of UC include 5-aminosalicylic acid drugs, corticosteroids, immune-suppressants, and biological therapy [1]. Biologic therapies, mainly including anti- tumor necrosis factor-alpha (TNF-α) monoclonal antibodies, anti-integrin monoclonal antibodies, interleukins (IL)-12/IL-23 antagonists and others, demonstrated efficacy in managing moderate to severe UC [12]. These targeted therapies aim to modulate specific components of the immune system, thereby reducing inflammation and preventing disease progression. Despite expanding therapeutic options, 10-20% of patients still require proctocolectomy due to medically refractory disease [9].

In addition to conventional therapies, there is growing interest in the potential of microecologics, fecal microbiota transplantation, and herbal therapies as adjunct treatments for UC. For instance, herbs such as *Aloe vera* (L.) Burm.f. (Asphodelaceae, aloe) and *Prunus humilis* Bunge (Rosaceae, pruni semen) have been effective in repairing the physical and immune barriers. These herbs increase mucin expression, promote the abundance of beneficial bacteria, and upregulate sIgA-related gene expression in dextran sodium sulphate (DSS)-induced colitis rats [13, 14]. These interventions may offer alternative or complementary therapeutic options for UC patients, particularly those seeking natural remedies or experiencing adverse effects from conventional medications.

This article first reviews recent findings on the mechanisms of UC, including genetic susceptibility, immune cell dynamics and cytokine regulation, and gut microbiota alterations. Secondly, it also discusses current applications of biologic therapy, herbal therapy, microecologics, and fecal microbiota transplantation (FMT), along with their prospects and challenges.

## Molecular insights into UC

Both Crohn’s disease (CD) and UC are major types of IBD. Typical clinical features of CD include abdominal pain, diarrhea, weight loss, and fatigue. In CD, intestinal inflammation is segmental, asymmetric, and transmural, often affecting the terminal ileum and colon. The inflammation involves the entire thickness of the intestinal wall [15]. UC’s inflammation is characterized by infiltration of immune cells into the mucosa, as well as epithelial cell damage and ulceration, while the inflammation typically limited to the mucosal layer of the colon, which distinguishes it from CD. And UC is believed to result from the interaction between genetic susceptibility, immune factors, and gut microbiota alteration, leading to abnormal mucosal immune responses and impaired epithelial barrier function.

### Genetic markers and susceptibility in UC

Advances in genetic research have significantly enhanced our understanding of UC’s pathogenesis, revealing crucial insights into the genetic factors that contribute to disease risk and progression. Genome-wide association studies (GWAS) have identified numerous genetic loci associated with UC, although these findings are predominantly derived from studies of European populations. Variants in genes such as those within the Human leukocyte antigen (HLA) region, including specific single nucleotide polymorphisms (SNPs), have been linked to increased susceptibility to UC. Additionally, the presence of perinuclear antineutrophil cytoplasmic antibodies (ANCA) has emerged as a potential biomarker for disease activity. This chapter examines the genetic underpinnings of UC, highlighting the significance of identified risk loci, the role of genetic variations in immune-related pathways, and the implications for understanding the disease’s complex etiology.

#### Familial and genetic risk factors in UC

Genetics play a significant role in the susceptibility to UC. The variations in the strength of familial IBD and future risk of IBD in first-degree relatives support differences in genetic predisposition. Adjusted risks for UC and CD were reported to be significantly higher in first-degree relatives of IBD patients than in those without IBD, and familial risks were highest in twins, followed by nontwin siblings, and then offspring of affected parents [16, 17]. Familial risk was generally higher within generations (sibling-sibling) than between generations (parent-child), and familial risk increased as the number of affected first-degree relatives increased [16, 17]. The highest incidence was observed in pediatric IBD, pediatric-onset CD and pediatric-onset UC [17]. Studies have reported a slight male predominance in UC, whereas CD exhibits a more pronounced male predominance [18].

#### GWAS and genetic variants in UC

GWAS have been conducted to identify genetic variants associated with UC. Over 200 loci linked to IBD have been discovered through GWAS. But they are primarily derived from Caucasian IBD patients of European. Of these loci, 41 are specific to CD, 30 are specific to UC, and 137 are shared between both diseases [19–22]. However, nearly half of the IBD-associated loci have also been linked to other immune-mediated conditions, such as psoriasis and ankylosing spondylitis [19, 23]. The putative genes identified through these studies play various roles in gut immunity, including regulating the gut mucosal barrier, autophagy, epithelial restitution, microbial defense, and adaptive immunity [24, 25]. Smillie et al. [26] found in UC patients, some cell subsets were enriched for the expression of several GWAS associated risk genes, with M-like cells having the highest expression of GWAS associated risk genes, including *CCL20*, *NR5A2*, *JAK2*. *CCL20* expression correlated with Treg cells frequency in each sample. M-like cells had the largest module of predicted risk genes and were enriched for endocytosis and Th17 differentiation genes. These findings suggest M-like cells dysfunction may play an important role in the disease. Key genes identified through GWAS include those regulating mucosal immunity, autophagy, and microbial defense. For instance, SNPs like rs3131621, rs9275596, and rs11244 in the HLA region are associated with increased primary sclerosing cholangitis (PSC) risk and are crucial in distinguishing patients with UC-PSC from patients with UC alone [27]. Other variants, such as those in *CARD9*, also play a role in UC susceptibility. *CARD9* is associated with both CD and UC, and in a pediatric IBD study, there were nine variants around the locus containing *CARD9* [28]. The variants in HLA genes, such as HLA-A and HLA-C, were closely associated with UC, but did not reach genome-wide significance in this study.

#### The role of HLA Class II molecules and other genetic markers in UC

HLA class II molecules are normally expressed only on specialized immune cells and consist of chains encoded by three genes (HLA-DP, HLA-DQ, and HLA-DR) [29]. Both chains HLA-DP and HLA-DQ are polymorphic. However, HLA-DR is polymorphic only for DRB, with 4700 known alleles at population level [29]. HLA class II molecules are critical in UC susceptibility. Specific SNPs and haplotypes in the HLA region, such as HLA-DPA1^∗^01:03-DPB1^∗^04:01 (HLA-DP401) and HLA-DPA1^∗^01:03-DPB1^∗^03:01 (HLA-DP301), were associated with increased risk and potential protection, respectively [29]. NKp44^+^NK cell-mediated destruction of the intestinal epithelium in UC, and HLA-DP401 can engage NKp44 and activate NKp44^+^NK cells, mediating damage to intestinal epithelial cells (IECs) in an HLA-DP haplotype-dependent manner. Perinuclear ANCA levels were higher in UC compared to CD, and ANCA titer was associated with disease activity in UC [16, 30, 31]. Additionally, serum ANCA-IgG levels correlated with UC severity [32]. UC patients more frequently had at least two ANCA specificities compared to CD patients [30]. The sensitivity of PR3-ANCA was comparable to that of atypical perinuclear ANCA, but its specificity was significantly higher, suggesting that PR3-ANCA could serve as a potential biomarker for extensive UC [16, 33, 34].

Additionally, a high-salt diet promoted DSS-induced IBD progression by enhancing *RIPK3*-dependent necroptosis in colonic epithelial cells [35]. *RIPK3* expression was significantly upregulated in the human normal colon epithelial cell line NCM460. Furthermore, *RIPK3*^-/-^ mice exhibited severe IBD symptoms, suggesting that *RIPK3* is significantly associated with epithelial necroptosis. *RIPK3*^-/-^ mice was more susceptible to DSS-induced colitis.

#### Ethnic variations in UC genetic susceptibility

Genetic susceptibility to UC varies among different ethnic groups, reflecting diverse genetic backgrounds and environmental interactions. For instance, a Mendelian randomization study [36] investigating genes associated with rheumatoid arthritis (RA) and IBD in the East Asian population found that RA patients had a higher incidence of CD but a lower incidence of UC. SNP rs2071475 is strongly associated with RA and exhibits opposing effects in CD and UC. Additionally, SNP rs2071475 is closely related to HLA-DOB, which is upregulated in CD but shows no significant changes in UC. Ashkenazi Jewish individuals have a 3 to 5 times greater risk of developing UC compared to other ethnic groups [37]. Genetic variations in genes such as interferon regulatory factor 5 (IRF5), TLR4, and vitamin D receptor (VDR) are linked to susceptibility across different populations [38–42], with specific SNPs like rs3807306 and rs4728142 associated with protective and risk factors, respectively [38]. Overall, these studies suggest that the related genes and genetic loci has increased our comprehension of the immune-mediated pathways involved in its pathogenesis and natural history.

### Immune cell dynamics and cytokine regulation in UC

UC is a multifactorial autoimmune disease characterized by a dysregulated immune response and impaired epithelial barrier [2, 43]. And immune dysfunction in UC involves both innate and adaptive immune systems. Although the mechanism is still unclear, immune cells such as T cells, B cells, and macrophages have been implicated in the pathogenesis of UC, as well as several cytokines and chemokines that regulate immune responses.

#### Immune activation and cytokine pathways in UC

UC is a disease that is characterized by dysfunctions in both the innate and adaptive immune systems. Antigens activate the innate immune response through antigen-presenting cells and T cells, triggering an inflammatory cascade that also activates the adaptive immune system [44, 45]. In the case of UC, mature dendritic cells become more sensitive and activated, indicating their significant involvement in generating inflammation [46]. Dendritic cells (DCs) in the innate immune system serve as antigen-presenting cells and play a role in initiating, regulating, and maintaining immune responses. DCs abundantly express Toll-like receptors (TLRs) that recognize pathogen patterns and activate several transcription factors, such as nuclear factor-κB (NF-κB), which trigger inflammatory cascades [47]. This process leads to the production of proinflammatory cytokines, notably TNF-α, IL-12 and IL-23 [44, 45]. These cytokines transmit messages via intracellular proteins like Janus kinases (JAK), which enhance lymphocyte activation and proliferation [48, 49]. Crypt scarring in UC results from inflammatory cell infiltration, including neo-lymphotropic and T-lymphocytes, causing damage to epithelial cells [2, 43, 50, 51]. IL-8, a potent neutrophil chemoattractant, increases neutrophil accumulation in crypts during acute phases, contributing to crypt destruction and correlating with UC severity [52] . Additionally, TNF-α, IL-12, and IL-23 can induce IL-8 production, highlighting their roles in mucosal protection and neutrophil recruitment [53–55]. Therefore, proinflammatory cytokines and intracellular proteins are the targets of many treatments for moderate to severe UC, including monoclonal antibodies to TNF-α and IL-12/23 receptors, which affect these pathways.

#### Role of CD4^+^ T cell subsets and cytokine imbalance in UC

UC patients exhibit infiltration of inflammatory CD4^+^ T cells in intestinal tissue [56]. CD4^+^ T cells differentiate into various subsets, including regulatory T (Treg) cells, T helper (Th)1 cells, Th2 cells, and Th17 cells [56, 57]. Th1 and Th2 cells are responsible for clearing intracellular and extracellular pathogens, respectively. In contrast, Th17 cells can induce autoimmunity and contribute to tissue damage [58]. Conversely, Treg cells can suppress tissue damage caused by immune and inflammation [59].

Th17 cells mainly secrete the pro-inflammatory cytokines IL-17A, IL-17F, IL-21, and IL-22, while Treg cells inhibit autoimmunity and secrete IL-10 [50]. The imbalance between Th17 cells and Treg cells was found to be a crucial factor in the pathogenesis of UC [60–62]. Serum IL-17 levels are significantly elevated in UC patients [63]. The number of Th17 cells and the mRNA expression levels of IL-17, IL-17A, IL-21, IL-22, and IL-23 in the inflammatory colonic tissues of active UC were significantly higher than those in inactive UC, healthy controls, and in vitro studies [63–66]. Additionally, the combined expression of IL-17A, IL-17F, IL-21, retinoic acid receptor-related orphan receptor C (RORC), and transforming growth factor (TGF)-β mRNA significantly predicted the rachmilewitz endoscopic index (REI), with IL-17A and IL-17F being associated with increased and decreased REI, respectively [67].

**Fig. 1:**
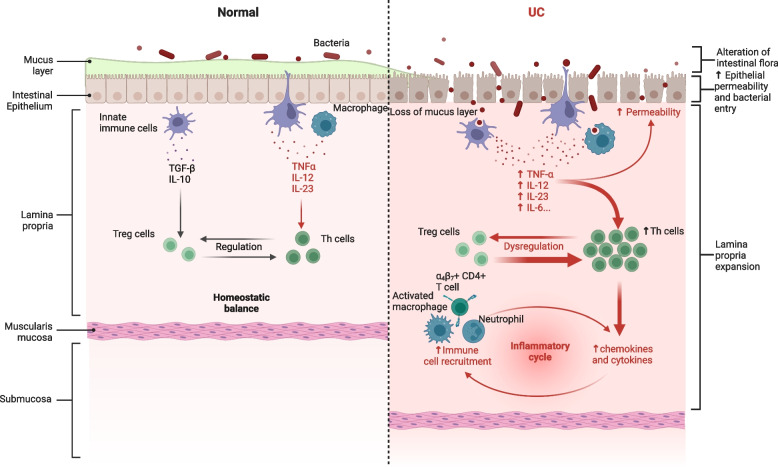
Potential immune response in UC. UC is a multifactorial autoimmune disease characterized by a dysregulated immune response, an impaired epithelial barrier, and alterations in the intestinal flora. In UC, increased intestinal permeability, pathogen invasion, and alterations in intestinal flora contribute to immune cell activation, an imbalance between Th and Treg cells, and elevated proinflammatory cytokine levels, which lead to further immune cell activation and worsening inflammation. ‘Adapted from “FullTemplateName”, by BioRender.com (CurrentYear). Retrieved from https://app.biorender.com/biorender-templates’

FOXP3 is a transcription factor essential for the development and function of CD4^+^CD25^+^ Treg cells. FOXP3^+^ CD4^+^ T cells were found to be increased in the lamina propria of both inflamed and non-inflamed areas in UC, compared to normal colon tissue [68]. And CD4^+^CD25^+^ T cells effectively inhibited colonic effector T cell activity and the production of Th1 (interferon (IFN)-γ, IL-2) and Th2 (IL-5, IL-13) cytokines in vitro, while the frequency of Treg cells in the inflammatory area increases with the increase of active UC disease activity [68, 69]. This may be because their inhibitory activity is disrupted in vivo or they are unable to counteract chronic mucosal inflammation in UC. There are conflicting findings regarding changes in the number and function of Treg cells in the gut and circulation of IBD patients. A 2024 meta-analysis of 17 studies (402 UC and 362 CD) [70] showed that the percentage of circulating Treg cells was significantly reduced and the inhibitory function of circulating Treg cells was impaired in patients with active IBD and healthy controls, and the proportion of Treg cells in the inflammatory area of the intestine was higher than that in the non-inflammatory area. However, compared with other intestinal inflammatory diseases such as intestinal tuberculosis, patients with IBD have a lower tendency to increase the proportion of intestinal Treg cells. The potential immune response in UC is shown in Fig. 1.

#### Regulatory B cells and IL-10 in controlling inflammation in UC

Regulatory B (Breg) cells can produce IL-10, which can prevent DCs from generating pathogenic T cells [71]. In patients with chronic inflammation, the immune system remains continuously activated, primarily due to a deficiency in the number and function of Breg cells in both the circulatory system and the sites of inflammation [72]. Innate CD5^+^ Breg cells negatively controlled innate inflammation and DC functions in neonatal mice by producing high amounts of IL-10 following TLRs triggering [73]. Breg cells are believed to regulate the induction of autoimmune disease by inhibiting the differentiation of Th1 and Th17 cells, while promoting the proliferation of Treg cells [74].

#### Cytokine-mediated leukocyte recruitment and barrier dysfunction in UC

Cytokines stimulate the expression of adhesion molecules on the vascular endothelium of blood vessels, promoting leukocyte adhesion and extravasation into the inflamed mucosa. As a result, circulating leukocytes are recruited to the site of inflammation, intensifying the inflammatory response. This process is largely mediated by cytokines [75]. Activation of T cells by α4β and mucosal addressin cell adhesion molecule-1 (MadCAM-1) facilitates their interaction in the mesenteric lymph nodes, entry into the bloodstream, and migration to the lamina propria of the mucous membrane [76]. Upon encountering antigens upon returning to the gut, primarily bacteria, these T cells release cytokines that aid in pathogen elimination [2, 43, 50, 77]. In the local colonic mucosa of UC patients, IL-9 and IL-13 levels are specifically increased [50]. IL-9 from Th9 cells and IL-13 from Th2 cells potentially increase neutrophil migration [53] and mucosal permeability [78], exacerbating barrier dysfunction.

#### The dual role of IL-22 in UC

IL-22 could be produced by immune cells like Th17 cells on mucosal surfaces, and its role in maintaining intestinal barrier homeostasis is a topic of debate.

Adjacent to the mucosal barrier, three groups of innate lymphoid cells (ILCs)-ILC1s, ILC2s, and ILC3s-secrete effector cytokines that regulate UC pathology [79]. ILC3s respond to IL-1β, IL-23, IL-6, IL-2, and IL-7, producing IL-22, IL-17, and TNF-α. UC patients show increased ILC1s and ILC2s frequencies but decreased NKp44^+^ ILC3s, which are crucial for IL-22-mediated epithelial barrier protection [80].

Recent research highlighted the significant role of long noncoding RNA (lncRNA) in the occurrence and progression of IBD [81, 82]. For example, lncRNA uc.173 promotes intestinal mucosal renewal by inducing the degradation of MicroRNA 195 [83]. IL-22 has been shown to alleviate DSS-induced colitis by upregulating lncRNA-UCL expression [84]. The mechanism involves regulating tight junction proteins (TJs) such as claudin-1, ZO-1, and E-cadherin, as well as the lncRNA/miR-568/claudin-1 axis, which is crucial for the normal growth of IECs [84]. Specifically, IL-22 upregulates lncRNA-UCL, enhances claudin-1 expression by sequestering miR-568, reduces pro-inflammatory cytokines (TNF-α, IL-1β, IL-6, IL-17A), and thus promotes IEC proliferation while preventing apoptosis [84].

Additionally, the *Citrobacter rodentium* (*C. rodentium*)-induced colitis model is valuable for studying epithelial dysfunction in IBD [85]. *C. rodentium* infection can damage colonic epithelial cells, potentially leading to epithelial hyperplasia [86]. IL-22 has been shown to mitigate intestinal injury in *C. rodentium*-infected FVB mice. This effect may be mediated by inhibiting Wnt-driven epithelial proliferation and dysfunction, and by correcting solute transport disorders that control the physiological balance of the Na^+^/Cl^-^ (decreased fecal Na^+^ and Cl^-^ levels, increased serum Na^+^ and Cl^-^ levels), and reducing serum total protein [87–89]. This restores Wnt signaling defects in IECs, prevents hyponatremia, hypochloremia, and dehydration, and normalizes intestinal physiological functions in *C. rodentium*-infected FVB mice [89]. Furthermore, IL-22-induced claudin-2 upregulation can drive diarrhea and pathogen clearance [90]. While claudin-2 upregulation promotes *C. rodentium* clearance and limits mucosal immune activation and tissue damage in rodents, it can also exacerbate disease symptoms. The severity of DSS colitis increased in claudin-2 knockout *C. rodentium*-infected mice, with elevated expressions of IL-1β, IL-6, IL-22 and TNF-α compared to wild-type or transgenic mice [90]. IL-22 selectively and significantly upregulated claudin-2 mRNA expression in organoid cultures and *in vivo* in the colonic epithelium. Additionally, elevated IL-22 levels were observed 2 days post**-***C. rodentium* infection, aligning with IL-22-dependent upregulation of claudin-2. It is hypothesized that epithelial NF-κB signaling activates subepithelial immune cells to produce IL-22 following *C. rodentium* infection [91, 92], potentially involving the IL-22RA signaling pathway in the intestinal epithelium [93].

Nevertheless, IL-22’s role in chronic colitis and its association with resistance to ustekinumab treatment underscores its dual nature [94]. The IL-22-responsive transcriptional module is significantly enriched in both mouse colitis models and human UC, with elevated levels of neutrophil-active chemokines such as *Cxcl1*, *Cxcl2*, *Cxcl3*, and *Cxcl5* [94]. IL-22-mediated regulation of CXCR2^+^ neutrophils is a crucial pathogenic pathway in UC, suggesting that the core chemokine module is conserved in colitis development, possibly through signal transducer and activator of transcription 3 (STAT3) signaling [94]. Tofacitinib, a selective JAK1/JAK3 inhibitor that prevents STAT3 phosphorylation and activation, has been approved for UC treatment [95]. Tofacitinib also significantly inhibits IL-22-induced CXCL1 and CXCL5 expression in human colonic tissues, suggesting that targeting the IL-22/CXC chemokine axis through JAK/STAT inhibition may be a viable therapeutic strategy.

#### IL-6 regulation and therapeutic targeting in UC

IL-6 involves in the pathogenesis of autoimmune diseases by modulating the balance between Th17 and Treg cells [96]. Rhbdd3, a member of the rhomboid protease family, negatively regulates DC activation and maintains the balance of Treg and Th17 cells by inhibiting IL-6 production in DCs, thus contributing to the prevention of autoimmune diseases [97]. Rhbdd3 inhibits DC activation and TLR-induced IL-6 production, reduces Th17 cell subsets, and increases Treg cells [98, 99]. Mechanistically, Rhbdd3 directly binds to the Lys27 (K27)-linked polyubiquitin chain on Lys302 of the regulatory factor NEMO (IKKγ) via its ubiquitin-binding (UBA) domain in the endosome. Rhbdd3 further recruits the deubiquitin enzyme A20 through the K27-linked polyubiquitin chain on Lys268, inhibiting K63-linked NEMO polyubiquitination. This process inhibits TLR-induced DC activation, subsequently inhibiting NF-κB activation and IL-6 production [97].

Furthermore, IL-6 has multiple functions, among them its ability to regulate immune responses. It can be produced by various cells in the human body and recognized by both transmembrane and soluble receptors [100]. The IL-6 cytokine and its receptor have garnered significant attention as important therapeutic targets [101, 102]. The IL-6 receptor complex consists of two subunits, IL-6 receptor α (IL-6Rα) and glycoprotein 130 (gp130). IL-6Rα is a non-signaling subunit that binds exclusively to IL-6. Additionally, IL-6 can initiate signaling through soluble IL-6Rα, expanding the range of responding cell types [103], and the primary downstream pathway activated by is the JAK/STAT pathway [104]. Groza et al. [105] explored the potential of IL-6Rα as a target for UC, and they selected a series of protein variants called NEF ligands from combinatorial libraries derived from albumin-binding domain scaffolds. These NEF conjugates demonstrated binding specificity to human IL-6Rα, and can recognized maturation-induced IL-6Rα expression and interferes with IL-6-induced differentiation of human primary B cells [105]. Among these, NEF108 significantly ameliorated the symptoms and pathological changes of DSS-induced colitis and reduced DSS-induced IL-1β expression, suggesting that IL-6R is a potential therapeutic target in UC [105].

#### Activation of NF-κB signaling pathway aggravates the inflammation of UC

The NF-κB signaling pathway is a central regulator of inflammation. In UC, the activation of NF-κB leads to the transcription of pro-inflammatory genes, perpetuating the inflammatory response [106]. In unstimulated cells, NF-κB dimers are usually inactive and held in the cytoplasm by small inhibitory molecules from the NF-κB inhibitory proteins family [107]. IKK activation could phosphorylate NF-κB inhibitors, leading to their degradation by the proteasome, which then activates and releases NF-κB complexes into the nucleus to trigger the expression of target genes involved in cell proliferation and apoptosis, initiating downstream signaling pathways [108]. And increased expression of NF-κB P-p65 expression was associated with the intestinal mucosal inflammation severity in UC models [109].

**Fig. 2:**
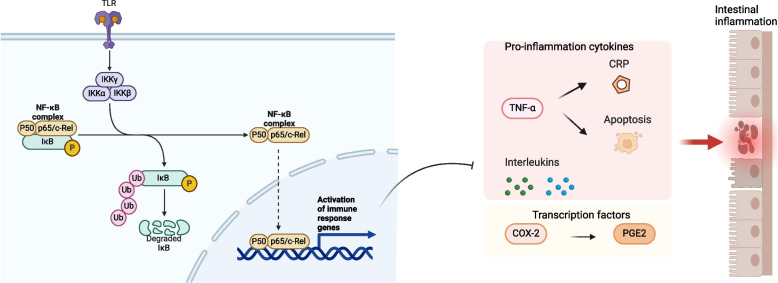
Activation of NF-κB signaling pathway increases the expression of pro-inflammatory genes to aggravate the inflammation of UC. IKK activation can phosphorylate NF-κB inhibitors, leading to their proteasomal degradation. This process activates and releases NF-κB complexes into the nucleus, triggering the expression of target genes involved in cell proliferation and apoptosis, and initiating downstream signaling pathways. Increased expression of NF-κB P-p65 is associated with elevated secretion of proinflammatory cytokines and other factors, exacerbating intestinal mucosal inflammation in UC. ‘Adapted from “FullTemplateName”, by BioRender.com (CurrentYear). Retrieved from https://app.biorender.com/biorender-templates’

NF-κB further contributes to the inflammatory response by controlling the levels of inflammatory factors such as IL-6 and TNF-α [106, 110]. IL-6, TNF-α, and IL-1β are key inflammatory cytokines involved in UC inflammation [111–114]. COX-2 catalyzes the synthesis of prostaglandins (PG) from arachidonic acid. In UC, COX-2 expression is elevated in the mucosal epithelium and crypt, where it increases PGE2 expression through the NF-κB signaling pathway. This leads to enhanced vasodilation, increased permeability, and mucosal congestion and edema, resulting in abdominal pain and diarrhea [115] (Fig. 2).

#### JAK/STAT signaling pathway can regulate UC inflammation by inflammatory cytokines

The JAK/STAT signaling pathway transmits extracellular signals to the nucleus, affecting gene expression and cellular responses. It coordinates intracellular signaling for over 50 different cytokines and consists of three members: tyrosine kinase-related receptors, JAKs, and STATs [116]. The JAK family (JAK1, JAK2, JAK3, TYK2) binds non-covalently to cytokine receptors, while STATs, as JAK substrates, couple with the tyrosine phosphorylation signaling pathway [117]. JAKs can link to multiple STATs, with STATs being the final effectors of signaling, and the combinations can be activated to regulate transcription, mediating processes like apoptosis, proliferation, differentiation, and cell migration [117, 118]. Dysregulation in this pathway can lead to severe immunodeficiencies, and its role extends to pro-inflammatory signaling mechanisms [119].

**Fig. 3:**
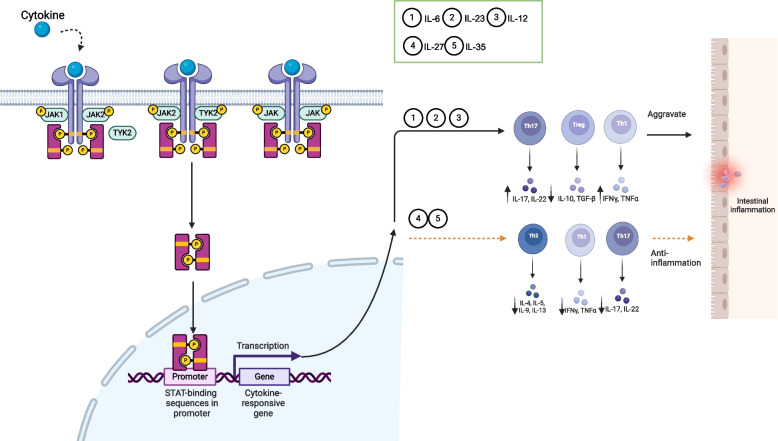
JAK/STAT signaling pathway can be activated or inhibited by inflammatory cytokines to regulate the inflammation of UC. The JAK family (JAK1, JAK2, JAK3, TYK2) binds non-covalently to cytokine receptors, and STATs, as JAK substrates, interact with the tyrosine phosphorylation signaling pathway. JAKs can interact with multiple STATs, which act as the final effectors of signaling, and these combinations can be activated to regulate transcription. Cytokines, including IL-6, IL-12, IL-23, and IL-27, activate the JAK/STAT signaling pathway via the JAK family, regulating the release of Th and Treg cells, thereby influencing UC inflammation. ‘Adapted from “FullTemplateName”, by BioRender.com (CurrentYear). Retrieved from https://app.biorender.com/biorender-templates’

Abnormalities in this signaling pathway can lead to defective T-cell differentiation and regulatory activity, which are crucial in the pathogenesis of UC [120]. Through the JAK family, IL-6, IL-12, IL-23, and IL-27 activate the JAK/STAT signaling pathway regulating the release of Th cells and Treg cells, which influence UC inflammation (Fig. 3) [62, 121–128]. IFN-γ produced by Th1 cell activation stimulates STAT1 in Th2 cells, affecting Th2 cells and the Th1/Th2 balance, thereby aggravating pathological changes in experimental colitis. Additionally, IFN-γ triggers *CLDN2* gene transcription, promoting claudin-2 expression and enhancing intestinal barrier permeability [129, 130].

Variants in JAK2, TYK2, STAT1, STAT3, STAT4 genes increase UC susceptibility [19], and JAK3 knockout mice develop severe combined immunodeficiency, affecting T and B cell development, resulting in IEC differentiation defects and impaired intestinal barrier function, thus increasing susceptibility to UC [131]. In addition, inhibiting STAT3 phosphorylation has been shown to restore the balance of Treg/Th17 cells in colonic tissue and alleviate UC symptoms in DSS-induced UC mice [62].

The intricate balance between pro-inflammatory and anti-inflammatory mechanisms is central to the development and progression of ulcerative colitis. Key cytokines, such as IL-6 and IL-22, along with regulatory immune cells, including Th17, Treg, and Breg cells, play significant roles in modulating the inflammatory response and maintaining mucosal integrity. Disruptions in these processes contribute to disease severity and highlight potential therapeutic targets. Advances in understanding these immune interactions offer promising avenues for novel treatments aimed at restoring immune balance and improving patient outcomes in UC. Continued research into these mechanisms is essential for developing targeted therapies and enhancing our ability to manage this challenging condition.

### Gut microbiota alterations and their impact on inflammation, nutrition, and immune responses in UC

Individuals suffering from UC experience imbalances in their gut microbiota, commonly referred to as “microbial dysbiosis”. Dysbiosis mainly refers to changes in the abundance and diversity of microbiota. The gut microbiota plays a crucial role in constituting the intestinal barrier, enhancing the immune response of the intestinal mucosa, and maintaining the intestinal environment. The gut microbiota imbalance can lead to intestinal barrier damage, and both intestinal barrier damage and gut microbiota imbalance are involved in the development of UC.

#### Differences in gut microbiota between UC patients and healthy individuals

The imbalance of intestinal flora UC is characterized by a decrease in bacterial diversity, especially in regions of active inflammation, specifically a reduction in the amounts and diversity of bacteria phyla Firmicutes and Bacteroides and an increase in the proportions of *Enterobacteriaceae* such as *E coli* [132].

There were no significant differences between inflamed and non-inflamed intestinal mucosa in UC, but UC was significantly different from CD and non-IBD mucosal gut microbiota [133]. Besides Bacteroidetes and Fusobacteria, Proteobacteria and Firmicutes were more common in the mucosa of UC at the phylum level. And at the genus level, the genera *Pseudomonas*, *Haemophilus*, and *Sporacetigenium* were found only in UC ileal mucosal samples [133]. Additionally, in UC samples there was an increase in *Coprococcus* and *Faecalibacterium* compared to healthy controls [133, 134]. Desulfovibrio bacterial species expression was significantly increased in the colon of patients with UC, especially in acute UC [135]. A 2020 meta-analysis (included 48 IBD studies) [136] identified that for UC patients, *Eubacterium rectale* and *Akkermansia* were decreased, whereas *E coli* was increased. And the diversity of gut microbiota in UC was reduced or unchanged compared with the control group [136].

Additionally, although inflammation in UC is primarily located to the colon, a small Swedish study [137] found reduced duodenal richness of Firmicutes, Actinobacteria, and Bacteroidetes in children with UC. Moreover, the detection rates of several bacterial genera including *Collinsella*, *Lactobacillus* and *Bacillus* were lower than those of non-IBD control group.

#### The decrease of SCFAs can lead to intestinal inflammation and the loss of nutrition

The most abundant short-chain fatty acids (SCFAs) in the human colon are acetate, propionate, and butyrate, can supply energy to cells in the colon, possess anti-inflammatory properties, and protect epithelial barrier integrity [138–140]. Acetate could directly activate the nucleotide-binding oligomerization domain containing 3 (NLRP3) inflammasome in mouse IECs, leading to the release of IL-18, and then promote the integrity of the intestinal barrier by activating the IL-18 receptor on mouse epithelial cells [141]. Butyrate could alter levels of claudin-2 in vitro or induce colonic mucin expression to maintain intestinal integrity [142]. Propionate can reduce intestinal permeability in UC mice by increasing the expression of ZO-1, occludin and E-cadherin [143]. SCFAs have the capacity to inhibit the NF-κB pathway [144–147] and the transcription of proinflammatory mediators, including TNF-α and IL-1β [148]. Studies have showed that the levels of SCFAs, especially n-butyrate, iso-butyrate, and acetate, in the intestinal mucosa and feces of UC patients were lower than those of healthy controls [149, 150]. This may be related to the reduction of butyric acid-producing members of clostridial cluster, such as *Clostridium coccoides* and *Clostridium leptum*, and the abundance of *Fecalibacterium prausnitzii* and *Roseburia intestinalis* was significantly associated with severe of UC [149, 150]. In addition, *Bifidobacterium* was increased in patients with active UC, and probiotics should be used with caution in active UC [150]. Consequently, it has been hypothesized that a reduction in SCFAs leads to heightened immune responses and epithelial nutrient deficiencies, which increase the risk of UC [151]. And the decrease of SCFAs in UC caused by the consumption of acetyl CoA by tricarboxylic acid cycle was higher in UC than in the controls [151].

#### Bacterial antigen exposure affects intestinal barrier function and immune response

Impaired mucosal function can increase mucosal permeability, and defective innate bacterial killing (*e.g.*, intracellular killing and secretion of antimicrobial peptides) can result in higher levels of bacterial antigens. This leads to abnormal immune regulatory mechanisms, which may activate pathogenic immune responses or invasive T cells with abnormal bacterial antigens and cause a loss of normal mucosal immune tolerance. In UC patients, there is reduced diversity of intestinal flora, altered types and quantities of intestinal flora, and increased infiltration of mucosal CD4+ T cells in the inflammatory areas. These factors contribute to abnormal immune responses due to defects in bacterial killing and dysregulated immune pathways [138, 152].

Antibiotics have been considered to treat UC due to their antimicrobial properties against intestinal bacteria linked to inflammation. Studies have demonstrated that although antibiotics do not play a significant role in treating UC in all trails, broad-spectrum oral antibiotic cocktails have reported a possible role in acute severe colitis and chronic persistent UC [153–156] .However, a systematic review of 12 randomized controlled trials (RCTs, include 847 participants) reported that no difference between antibiotics and placebo in the proportion of patients in clinical remission at the end of the intervention period [157].

## Intervention therapy

The current treatment of UC includes conventional therapies, biologic drugs, surgical treatment, complementary therapies, and others. Conventional therapies, including aminosalicylic acid (5-ASA), corticosteroids, and immunomodulators, play a crucial role in managing inflammation, initiating and sustaining remission, and enhancing the overall quality of life for patients [158]. 5-ASA is a fundamental component of treatment, with sulfasalazine being the main source of side effects. 5-ASA medications include sulfasalazine, olalazine, mesalazine, and basalazine. These drugs are widely recognized for their efficacy, safety, and affordability in the treatment of UC [159, 160]. Corticosteroids, such as prednisone and budesonide, are frequently used for moderate to severe UC, especially when 5-ASA is insufficient to manage symptoms [161]. Topical steroids, such as suppositories, foams, and enemas, work well for distal colitis. However, corticosteroids do not represent a therapeutic option as a maintenance treatment since they are associated with multiple adverse effects. Moderate UC treatment involves both corticosteroids and 5-ASA, while 5-ASA and corticosteroids are typically enough for mild UC cases. And if patients don’t respond to 5-ASA or corticosteroids, it is advised to use immunomodulators like azathioprine and 6-mercaptopurine to maintain remission [162]. The clinical feasibility and low toxicity of immunosuppressants are increasing their use in IBD therapy.

As drug development advances, the range of medications for UC expands. New therapies, such as biological agents, herbal therapies, and microecologics and FMT, have become significant research hotspots and are discussed in the following sections of this article.

### Biologic therapy

Biologic therapies are a relatively new class of pharmacologic interventions that specifically target specific molecules or cells involved in inflammatory pathways in UC. These therapies are generally recommended for patients with moderate-to-severe UC who do not respond to conventional treatments. Various biologics mainly include anti-TNF-α monoclonal antibody(mAb), anti-α4β7 mAb, IL-12/23 mAb, and JAK inhibitor, and they have been approved for moderate to severe UC, showing greater success than conventional treatments like 5-ASA and corticosteroids [163] (Table 1).

**Table 1:** Detailed information on the study of biological drugs

**Drug type**	**Drug name**	**Experimental type**	**Time point**	**Indicator**	**Group 1 (Dose)**	**Group 1 (Result)**	**Group 2 (Dose)**	**Group 2 (Result)**	**Placebo Result**	**Ref**
Anti-TNF-α mAb	Infliximab (IFX)	RCT	Week 8	Clinical remission	5 mg/kg	39%	10 mg/kg	32%	15% (*P* < 0.001, *P* = 0.002)	[164]
			Week 54	Sustained remission		20%		21%	7% (*P* = 0.002)	
		RCT	Week 8	Clinical remission	5 mg/kg	34%	10 mg/kg	28%	6% (*P* < 0.001)	[164]
				Mucosal healing rate		62%		59%	33.9% (*P <* 0.001)	
		Prospective clinical study		CRP and fecal calprotectin		Reduce				[165]
		Clinical study		Surgery rate		27% to 11%				[166]
	Adalimumab (ADA)	Retrospective, single-cohort, open-label study	Week 8	Histological remission		17.6%				[167]
				Mucosal healing rate		50%				
				Clinical response		85.3%				
				Clinical remission		23.5%				
			Week 52	Histological remission		31.0%		26.5%		
				Mucosal healing rate		72.4%		61.8%		
				Clinical response		79.3%		67.6%		
				Clinical remission		62.1%		52.9%		
		RCT	Week 8	Clinical remission	160/80 mg	18.5%	80/40 mg	10%	9.2% (*P*=0.031, *P*=0.833)	[168]
		RCT	Week 8	Clinical remission		16.5%			9.3%	[169]
			Week 52			17.3%			8.5%	
			Week 8	Mucosal healing rate		41.1%	-	-	31.7%	
			Week 52			25%	-	-	15.4%	
	Golimumab (GLM)	Multicenter, randomized, double-blind, controlled study	Week 30	Clinical remission	50 mg	23.2%	100 mg	27.8%	15.6% (*P* = 0.014, *P*<0.001)	[170]
			Week 54	Mucosal healing rate	50 mg	41.7%	100 mg	42.4%	26.6% (*P* = 0.011, *P* = 0.002)	
		RCT	Week 6	Clinical remission	200/100 mg	17.8	400/200 mg	17.9	6.4% (*P* < 0.0001 for both)	[171]
				Mucosal healing rate		42.3		45.1	28.7% (*P* = 0.014, *P* < .0001)	
Anti-integrin mAb	Vedolizumab(VDZ)	Clinical study	Week 6	Clinical response rate	300 mg	47.1%			25.5% (*P* < 0.001)	[172]
				Clinical remission		16.9%			5.4% (*P* = 0.001)	
		Clinical study	Week 52	Clinical remission	Every 8 weeks	41.8%	Every 4 weeks	44.8%	15.9% (*P* < 0.001 for both)	[173]
				Mucosal healing		51.6%		56%	19.8% (*P* < 0.001)	
		Clinical study	Week 52	Deep remission		27%		28%	8.7% (*P* < 0.0001)	[174]
	Etrolizumab (ETZ)	Phase III trial	Week 62	Endoscopic remission		31%			17% (*P* = 0.029)	[175]
IL-12/IL-23 mAb	Ustekinumab (UST)	RCT	Week 8	Clinical remission	130 mg	15.5%	6 mg/kg	15.6%	5.3% (*P* < 0.001 for both)	[176]
				Mucosal healing rate		20.3%		18.4%	8.9% (*P* < 0.001 for both)	
		Real-world study	Week 16	Clinical response		53%				[177]
				CRP		52%			75% (*P* < 0.05)	
				Endoscopic activity		50%			74% (*P* < 0.05)	
			Week 52	Clinical remission	every 12 weeks	38.4%	every 8 weeks	43.8%	24.0% (*P* = 0.002, *P* < 0.001)	[178]
JAK inhibitor	Tofacitinib (TFT)	Phase III trial	Week 8	Clinical remission		16.6%			3.6%	
			Week 52			34.3%			11.1%	
		Real-world study		Adverse reactions		15.7% (5.8% serious, 4.2% discontinued)				[95]
	Filgotinib (FGT)	Phase 2b/3 study	Week 10	Clinical remission	200 mg	26.1%			15.3% (*P* = 0.0157)	[179]
	Upadacitinib (UPT)	Randomized trial	week 8	Clinical remission	45 mg (induction therapy)	26 %			5% (*P*<0.0001)	[180]
		Phase III trial	Week 52	Clinical remission	30 mg (Maintenance therapy)	52%			12% (*P*<0.0001)	[181]

The primary time point for short-term observation was the eighth week. Key indicators included clinical remission, mucosal healing rate, histological remission, clinical response, as well as CRP and fecal calprotectin levels

#### Anti-TNF-α mAb

TNF-α is a crucial inflammatory cytokine involved in mediating inflammation in UC patients. It triggers other proinflammatory factors, activates macrophages and T cells, and leads to epithelial cell injury and intestinal mucosal damage. Increased levels of TNF-α can be found in the colon, blood, and feces of UC patients. Currently, anti-TNF-α mAb approved for UC treatment include (Infliximab (IFX), adalimumab (ADA), golimumab (GLM)).

IFX is a chimeric human-mouse immunoglobulin (Ig) G1 mAb that binds to and neutralizes TNF-α. It is an approved biologic agent for UC and has the most reliable data for treating acute severe UC [182]. Research indicates that an 8-week continuous use of IFX significantly improves clinical symptoms, achieving higher clinical remission (*P* < 0.05) and mucosal healing rates (*P* < 0.001) compared to a placebo group [182]. This improvement may be linked to its ability to inhibit C-reactive protein (CRP) and fecal calprotectin levels, stimulate an immune response, indirectly regulate intestinal flora, and thereby enhance UC activity while reducing surgery rates [164, 183]. A 2021 meta-analysis found IFX to be the most effective among various biological agents for endoscopic improvement, although the 5 mg/kg dosage is associated with serious adverse events [12].

ADA, a fully human recombinant IgG1 mAb, binds to both soluble and membrane-bound TNF-α to induce cell-mediated cytotoxicity, complement activation, and T cell apoptosis, improving UC [184]. ADA is suitable for patients intolerant to IFX and has shown better clinical remission and mucosal healing in moderate to severe UC patients compared to the placebo group (*P* < 0.05) [167, 168], with demonstrated safety even during long-term use [185].

GLM is synthesized from transgenic mice immunized with TNF, and has a higher affinity for TNF than IFX and ADA, offering superior conformational stability and inhibition of TNF-induced cytotoxicity [186]. In a multicenter, double-blind RCT, clinical remission and mucosal healing at week 54 were significantly higher in patients receiving 100 mg of GLM compared to those receiving a placebo (*P* < 0.01) [170].

#### Anti-integrin mAb

After UC occurs, numerous white blood cells accumulate in the intestinal tract, leading to immune disorders. Integrins are transmembrane receptors on leukocyte surfaces, consisting of α and β subunits. The α4β7 integrin binds to MAdCAM-1 on intestinal endothelial cells, while the αEβ7 integrin binds to E-cadherin on mucosal epithelial cells. This interaction with memory T cells blocks inflammation, making integrins an ideal target for UC treatment [187]. Vedolizumab (VDZ) and Etrolizumab (ETZ) are representative anti-integrin mAbs.

VDZ, a humanized IgG1 mAb, selectively blocks the α4β7 integrin from binding to MAdCAM-1 on intestinal endothelial cells, preventing lymphocyte transport to the intestine and promoting mucosal repair [188–191]. This effectively alleviates symptoms in UC patients. For active UC, VDZ showed excellent clinical remission and reduced Disease Activity Index (DAI) compared to placebo (*P* < 0.001) [172], with the best safety outcomes [179].

ETZ is a mAb that inhibits both α4β7 integrin binding to MAdCAM-1 and αEβ7 integrin binding to E-cadherin [192]. In a phase III trial, ETZ achieved endoscopic remission in 31% of patients with moderate to severe active UC by week 62, significantly better than the placebo group (*P* < 0.05) [175].

#### IL-12/IL-23 mAb

Both IL-12 and IL-23 are composed of p40 subunits and others, and they are proinflammatory factors produced by enteric pathogens, essential for the differentiation of CD4 naive cells [193].

Ustekinumab (UST) is a fully human mAb that inhibits immune cell activation by targeting the p40 subunit shared by IL-12 and IL-23 on T cells, natural killer cells, and antigen-presenting cells [194]. In patients with moderate to severe UC, UST showed a better clinical remission rate during the maintenance phase, compared to placebo group (*P* < 0.001) [178], And UST inhibited the increase in CRP, the severity of endoscopic activity, and promoted mucosal healing in patients with clinical remission (*P* < 0.05) [177].

#### JAK inhibitor

The JAK-STAT signaling pathway regulates innate and adaptive immunity, enabling helper T cells to produce cytokines and induce inflammatory responses in UC [49]. The main small molecules that inhibit JAK are Tofacitinib (TFT), Filgotinib (FGT), and Upadacitinib (UPT).

TFT is a non-selective JAK inhibitor and mainly inhibits JAK1 and JAK3. and has been approved by the Food and Drug Administration (FDA) and European Medicines Agency (EMA) for the treatment of moderate to severe UC, which is the most widely used non-immunogenic oral small molecule drug in clinical practice [195]. TFT treatment in patients with moderate to severe UC resulted in a remission rate of 16.6% at 8 weeks, significantly higher than the placebo group (*P* < 0.001). TFT may normalize TJ expression, macrophage and PTPN2 functions in intestinal epithelial cells, as well as restore epithelial barrier integrity by inhibiting JAK signaling pathway activity [196].

FGT selectively inhibits JAK1 and has been approved for the treatment of UC through inducing and maintaining clinical remission. Patients tolerated FGT treatment well [197]. Nasopharyngitis and headache were the most common adverse events, but the adverse event rates were similar in the treatment and placebo groups.

UPT has a strong inhibitory effect on JAK1, achieving a significantly higher clinical remission rate than the placebo group (*P* < 0.0001). Patients tolerated UPT well during induction and maintenance therapies, and there were no new safety risks [180, 181]. In several meta-analyses, UPT was found to be the most effective small molecule drug for inducing clinical remission and clinical response, but it had the highest incidence of adverse events in patients with moderate to severe UC [12, 179]. Although it has been considered the best treatment in many analyses, these trials have not been fully published or rigorously peer-reviewed.

### Herbal therapy

Herbs and herbal ingredients are used as complementary and alternative therapies all over the world for the treatment of various diseases. Herbs can play a role in treating UC, by regulating inflammatory cytokines, intestinal flora, and the immune system, and protecting the intestinal mucosa [198]. Additionally, irritable bowel syndrome (IBS) is a common functional gastrointestinal disorder characterized by abdominal pain, abdominal discomfort and abnormal bowel habits, such as diarrhea or constipation, which are caused by changes in intestinal sensitivity and gastrointestinal motility [199]. UC patients in clinical remission usually experience IBS-like symptoms [200]. For instance, antispasmodic drugs (mainly anticholinergics) and antidiarrheal agents are used for abdominal pain and diarrhea. And anxiety and depression are frequent comorbidities in patients with IBS, and they may need use antidepressants [199]. Complementary therapies, such as herbal treatments, may also be beneficial for some individuals to alleviate these symptoms [199].

**Fig. 4:**
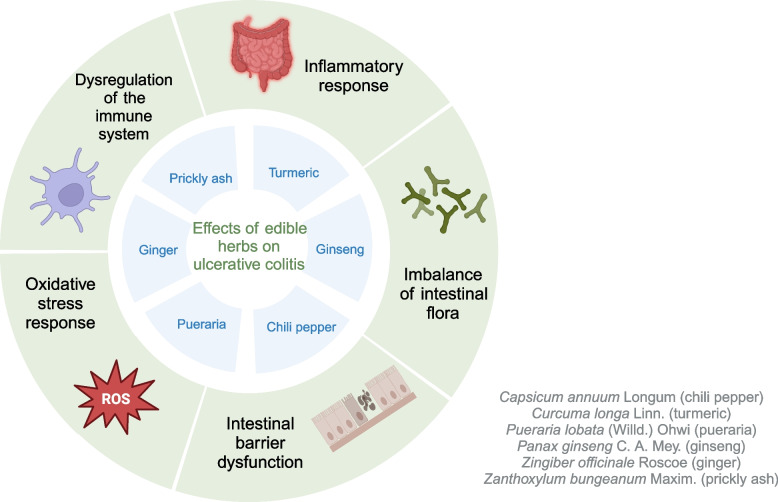
Effects of edible herbs on the UC. These edible herbal medicines have shown their efficacy in UC studies, potentially through mechanisms involving the regulation of inflammatory and immune responses, gut microbiota, oxidative stress, and intestinal barrier function. ‘Adapted from “FullTemplateName”, by BioRender.com (CurrentYear). Retrieved from https://app.biorender.com/biorender-templates’

The theory of homologous between medicine and food comes from traditional Chinese medicine (TCM), which means that even food can fulfill the purpose of medicine in treating diseases [201]. Such herbs seem safe to use, as they are themselves used as food and can be consumed daily, and we hypothesized that UC patients using them as food may have a positive effect on the disease. In this paper, six representative herbs with both medicinal and edible properties were selected, and the potential roles of them and their components in UC are described below (Fig. 4). Additionally, according to traditional medical theory, herbs are categorized by their “nature” (cold, hot, warm, cool), “flavor” (pungent, sweet, bitter, sour, salty), and primary functions.

#### Warm and hot herbs

In traditional medical theory, warm and hot herbs are used to expel cold and warm the body. *Capsicum annuum* Longum, commonly called chili pepper, is a perennial herb belonging to the genus Capsicum in the Solanaceae family. Due to their adaptability to cultivation and their recognized flavor, nutritional value, and potential health benefits, chili pepper holds a significant position in the culinary and food industries worldwide. Experiencing a burning sensation in the mouth and gastrointestinal tract is a common response when consuming chili pepper or spicy foods. Consequently, there is a prevalent belief that chili pepper can potentially induce or exacerbate gastrointestinal inflammation. Nevertheless, epidemiological studies conducted in various regions of China have discovered that areas with higher chili pepper consumption exhibit lower incidence rates of IBD compared to regions with lower chili pepper consumption [202, 203]. This finding contradicts the common belief that chili peppers may increase gut inflammation.

**Fig. 5:**
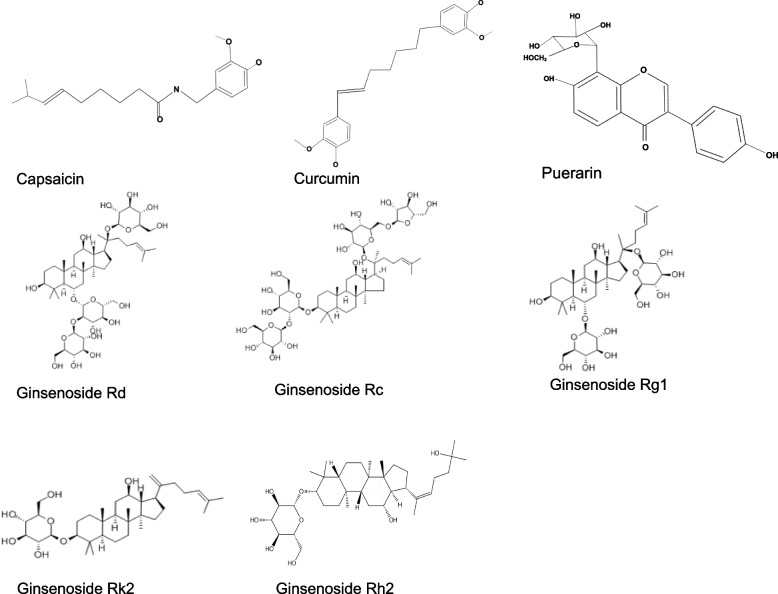
Chemical structures of capsaicin, curcumin, puerarin and ginsenosides. ‘Created with BioRender.com’

The main active ingredients in chili pepper are capsaicin and dihydrocapsaicin, and the total amount of capsaicin and dihydrocapsaicin measured in dried chili was at least 0.16% (2:1) [204, 205]. Capsaicin is also known as a common food additive, and the chemical structure of capsaicin is shown in Fig. 5. Capsaicin has been extensively studied, among which it has been confirmed to have positive effects related to UC. Capsaicin administered orally (12 mg/kg) or via enema (100 μg) reduced DAI and histopathological damage, and improve the general symptoms in the colon of rats with DSS-induced colitis [206, 207]. The mechanism may be the anti-inflammatory effect through the regulation of serum pro-inflammatory cytokines (such as IL-6) and anti-inflammatory cytokines (such as IL-10), and the anti-oxidative effect mediated by serum superoxide dismutase (SOD), catalase (CAT) and myeloperoxidase (MPO) [207].

Appropriate intake of capsaicin is helpful to relieve intestinal pain caused by UC. Transient receptor potential vanilloid cation channel subtype (TRPV1) and transient receptor potential ankyrin (TRPA1) play a crucial role in visceral sensation and pain [208]. Capsaicin can reduce the protein expression of TRPV1 andTRPA1 in the colon of DSS-induced UC rats [207]. And capsaicin, also known as a major agonist of TRPV1, activates sensory neurons and produces a burning pain sensation. High concentration of capsaicin exposure can make capsaicin play a neurotoxin role, triggering the internalization and degradation of TRPV1, thereby reducing the body’s sensitivity to pain and relieving the symptoms of the disease. Additionally, it inhibits intestinal Cl^-^ secretion and primarily enhances Na^+^ absorption by blocking TRPV4 channels in transgenic mouse models, including TRPV1- and TRPV4-knockout mice [209]. These results suggest that capsaicin may also relieve diarrhea and other symptoms of UC by reducing intestinal osmolality through regulating ion transport.

However, some studies have reported that capsaicin can exacerbate the severity of experimental UC [210, 211]. To investigate the role of TRPV1-expressing neurons in oxazolone-induced colitis, mice were subcutaneously injected with capsaicin (50 mg/kg) twice on day 2 and 5 after birth. Capsaicin can induce cell death in neurons with unmyelinated fibers (C-fibers) located in peripheral sensory ganglia [212]. Neonatal capsaicin treatment, resulting in sensory denervation, exacerbated colitis with increased weight loss, delayed recovery of body weight, and excessive neutrophil accumulation [210, 211]. Interestingly, these findings contradict the results of a previous study where newborns were given a subcutaneous dose of 50 mg/kg capsaicin on their first day of life [206]. The above results suggest that different doses, frequency of administration, or method of administration of capsaicin may be the reasons for the differences in conclusions.

Furthermore, studies reported that sugar consumption is positively associated with UC [213–215]. Sucrose could increase intestinal permeability and susceptibility to the development of colitis in mice, which may be mediated by increasing inflammatory cytokines (*e.g.*, IL-6, IL-1β, TNF-α, and PGE2 )and serum lipopolysaccharides levels [216–220] and disrupting the intestinal environment, including reducing the diversity of intestinal microorganisms [216, 217] and fecal SCFAs [216, 217, 221], and regulating intestinal PH value [221] and oxidative stress through glycoxidation (NO, protein carbonyl (PCO), MPO and alkaline phosphatase (ALP)) [220, 221]. In addition, sucrose easily promoted colon cell proliferation, which is considered to be a risk factor for colon tumors to some extent [218, 219]. The negative effects of sugars on UC are well recognized, and inhibiting the transport and utilization of sugars in the body seems to improve this situation. For instance, glucose serves as the primary energy source for activating inflammatory cells, and intracellular glucose uptake relies primarily on glucose uptake transporters (GLUTs), which serve as off-target molecules for ritonavir [222]. Ritonavir was able to alleviate colon pathological changes in immunocompromised NOD-scid IL-2Rγ^null^ mice reconstituted with peripheral blood mononuclear cells from UC patients. Moreover, the levels of M2 monocytes and Glu were reduced compared to the control group. Thus, inhibiting glucose uptake represents a potential therapeutic approach to inhibit inflammatory activation in UC.

Capsaicin plays an important role in modulating glucose homeostasis through mainly TRPV1 dependent and TRPV1 independent pathways [223], which means capsaicin has the potential to inhibit the negative effects of sugars in the body. Vascular endothelial senescence is primarily attributed to hyperglycemia. And capsaicin was reported to upregulate Sirtuin 1 (SIRT1) levels through TRPV1/[Ca^2+^]i/Calcium-calmodulin-dependent protein kinase II (CaMKII)/AMP-activated protein kinase (AMPK) pathway and inhibit intermittent high glucose-mediated endothelial cell senescence [224]. Oxidative stress and the resultant hyperpermeability play a vital role in the pathogenesis of diabetic retinopathy. Polymerase δ-interacting protein 2 (poldip2) has been implicated in H_2_O_2_ production. Liu et al. found that capsaicin could significantly activate TRPV1 and inhibit poldip2 and NADPH oxidase (Nox)4 under high glucose environment. The expression of peroxisome proliferator-activated receptor gamma (PPARγ), a nuclear receptor that down-regulates inflammation, is impaired in colonic cells of UC. Subsequently, they found that capsaicin can effectively inhibit the expression of vascular endothelial inflammatory mediators in both STZ-induced diabetic rats with diabetic retinopathy and human retinal microvascular endothelial cells exposed to high glucose. This inhibition is achieved by capsaicin’s interaction with the PPARγ-poldip2-Nox4 pathway [225].

Ginger, the rhizome of *Zingiber officinale* Roscoe in the Zingiberaceae family*,* is an essential edible herbal medicine and dietary supplement. Studies indicate that ginger exhibits a wide range of pharmacological activities, including anti-inflammatory [226], antioxidant [227], and antibacterial effects [228]. According to traditional usage, ginger is believed to have properties such as dispelling cold and relieving exterior symptoms, warming the middle-jiao to alleviate vomiting, dissolving phlegm, and suppressing cough. It has been extensively used for centuries to treat digestive disorders [205]. Currently, ginger shows promising potential as a treatment for UC in several experimental models and clinical trials [229–231].

In a clinical trial conducted on patients with active mild-to-moderate UC [229], it was discovered that taking 4 capsules of dried ginger powder daily (2000 mg in total) by 46 subjects resulted in a reduction in DAI scores and an improvement in their quality of life. This is because ginger has the potential to alleviate UC symptoms by reducing intestinal inflammation markers such as TNF-α and PGE2, decreasing oxidative stress markers including malondialdehyde (MDA) and PCO, modulating intestinal flora, and restoring the integrity of the intestinal barrier through proteins like occludin-1 and ZO-1 [229–231]. The administration of ginger extract at doses of 100, 200, and 400mg/kg resulted in a reduction in levels of oxidant stress factors such as MDA and PCO, as well as acute inflammatory markers including MPO, TNF-α, and PGE2, in rats with acetic acid-induced UC [230].

Polysaccharides are natural bioactive ingredients. Hao et al. [231] isolated polysaccharides from ginger, and the molecular weight, the radius of gyration and polydispersity of ginger polysaccharide (GP) were 7.472*105 Da, 1.929, and 47.7 nm, respectively. GP contained acidic polysaccharides and polysaccharide-protein complex, and 13.08% and 43.77% of uronic acid and protein were detected, respectively. Moreover, treatment with GP in mice with DSS-induced colitis resulted in the suppression of pro-inflammatory cytokines TNF-α, IL-6, IL-1β, IL-17A, and IFN-γ, modulation of intestinal barrier-related factors occludin-1 and ZO-1, enhancement of intestinal flora diversity, and an increase in *Lactobacillus* population [231].

Prickly ash is the dried and mature skin of *Zanthoxylum bungeanum* Maxim. in the Rutaceae family. Prickly ash is a widely used food additive and traditional herbal medicine. It has demonstrated anti-inflammatory [232], analgesic [232, 233], antioxidant properties [234], and regulatory effects on the gastrointestinal system [235, 236]. Studies have demonstrated that the *Zanthoxylum bungeanum* pericarp extract (ZBE) can alleviate colonic shortening, body weight loss, and disease activity index in mice with DSS-induced colitis [237–239]. The main flavonoids found in ZBE are rutin (32.36%), quercetin (13.61%), and isoquercitrin (24.89%). These compounds have been shown to reduce the levels of pro-inflammatory cytokines (TNF-α, IL-β, and IL-12), inhibit NF-κB p65 and IκBα phosphorylation, as well as modulate the TLR4 and TLR4-related pathways (NF-κB and MAPK) in vitro and in vivo [237]. In another study, it was found that the essential oil of ZBE suppressed TNF-α, IL-1β, and IL-12 levels both in vivo and in vitro, as well as NLRP3 inflammasome activation in vivo [238]. In vivo, it decreased the levels of NF-κB p65 and IκB phosphorylation while increasing the expression of PPARγ, which could potentially play an anti-inflammatory role in the progression of colitis [238]. Additionally, the essential oil of ZBE increased the levels of intestinal bacteria *Lactobacillus* and *Bifidobacteria*, which were reduced by DSS, while significantly decreasing the DSS-induced increase in *Escherichia coli* (*E. coli*) [238]. The expression of intestinal tight junction protein ZO-1 was also increased [238]. Furthermore, Zhang et al. [239] found the essential oil of ZBE treatment could decrease the expression of vascular cell adhesion molecule-1 (VCAM-1), TLR8, IL-1β and IL-11 mRNA, which are involved in these potential genetic pathways. Effects and potential mechanism of herbs on UC are summarized in Table 2.

**Table 2:** Effects and potential mechanism of herbs on UC

**Category**	**Potential mechanism**	**Effect on UC patients**
*Capsicum annuum* Longum	(1) It inhibits oxidative stress [207]. (2) It reduces the levels of inflammatory factors and increases the levels of anti-inflammatory factors [206, 207]. (3) It reduces the protein expression of TRPV1 and TRPA1 [207]. (4) It inhibits intestinal Cl^-^ secretion and primarily enhances Na^+^ absorption [209].	There was no clear evidence of an association with the risk of IBD [202, 203].
*Curcuma longa* Linn.	(1) It regulates inflammatory-and immune-related signaling pathways, such as TLR/MyD88, JAK1/STAT3/SOCS, TLR4/NF-κB/AP-1 signaling pathways [42–47]. (2) It affects the expression of immune cells [240, 241] and inflammatory cytokines [242, 243]. (3) It regulates intestinal flora [244].	It alleviates symptoms and prevents recurrence [245–248].
*Pueraria lobata* (Willd.) Ohwi	(1) It reduces the levels of inflammatory cytokines [249]. (2) It down-regulates the expression of Nrf2 pathway and antioxidant enzymes [249, 250]. (3) It activates AMPK/SIRT1 signaling pathway [251].	
*Panax ginseng* C. A. Mey.	(1) It reduces the levels of inflammatory cytokines in macrophages [252, 253]. (2) It elevates anti-inflammatory cytokines levels [254]. (3) It regulates the tryptophan metabolism [255]. (4) It regulates gut microbiome homeostasis [254, 255].	
*Zingiber officinale* Roscoe	(1) It reduces the levels of pro-inflammatory cytokines [230, 231]. (2) It reduces the levels of antioxidant factors [230]. (3) It increases the expression of intestinal TJs [230, 231]. (4) It improves intestinal flora [231].	It alleviates symptoms and quality of life [229].
*Zanthoxylum bungeanum* Maxim.	(1) It reduces the levels of pro-inflammatory cytokines [237–239]. (2) It increases the levels of anti-inflammatory cytokines [238, 239]. (3) It modulates the TLR4 and TLR4-related pathways (*e.g.*, NF-κB) [237, 238]. (4) It improves intestinal flora [238]. (5) It increases the expression of intestinal TJs [238].	

*Curcuma longa* Linn. and *Zingiber officinale* Roscoe may alleviate symptoms in UC patients. And these six herbs could potentially regulate inflammatory response, immune response, gut microbiota, oxidative stress and intestinal barrier function

#### Heat-clearing and detoxifying herbs

Heat-clearing and detoxifying herbs could clear heat and reduce inflammation in traditional medical theory*. Curcuma longa* Linn., known as turmeric, is a plant of the genus Curcuma in the Zingiberaceae family. Turmeric and other spices are combined to create curry, which is renowned as one of the world’s most popular sauces. Curcumin is the primary active ingredient and phenolic antioxidant in turmeric, and content of curcumin is at least 1% of the dried turmeric [205]. Curcumin’s chemical structure is shown in Fig. 5. It is widely recognized as one of the best-selling natural food colorings globally and has been approved as a food additive by the World Health Organization and the FDA.

Curcumin has shown potential in alleviating active UC symptoms and preventing recurrence during periods of quiescence [245–248]. For mild-to-moderate UC, oral administration of curcumin (1500 mg/day) can significantly reduce the scores on the Simple Clinical Colitis Activity Index, IBD Questionnaire-9, and quality of life of UC patients [256]. In another study of quiescent UC patients, the combination of curcumin (2 g/day) with sulfadiazine or mesalazine was able to reduce the recurrence rate, significantly reduce the clinical activity index (CAI) and significantly improve the endoscopic index (EI) [257]. Additionally, in UC patients, the levels of serum inflammation markers high-sensitivity CRP and erythrocyte sedimentation rate (ESR) were reduced [256]. And certain meta-analyses have summarized and analyzed these studies, indicating that adjuvant curcumin may be effective in achieving clinical remission of UC and holds therapeutic potential [258–260]. But one of the meta-analyses (included 385 patients) found the curcumin was ineffective in inducing endoscopic remission [258]. Non-standardized dosage forms, dosages, treatment timing, and administration routes in clinical studies contribute to the difficulty in assessing the outcomes and serve as the main reason behind controversial results [259, 260].

Curcumin could regulate multiple signal transduction pathways in experimental UC, including TLR/major myeloid differentiation response gene 88 (MyD88), JAK1/STAT3/SOCS, and TLR4/NF-κB/ activating protein (AP)-1, exhibiting anti-inflammatory, immunomodulatory and other effects [242, 261–265]. For instance, curcumin significantly reduced histopathological damage and improved general symptoms in experimental colitis, accompanied by increased the expression of Treg cells and decreased the expression of Th17 cells as well as increased the expression of anti-inflammatory cytokines such as IL-10 and decreased the expression of proinflammatory cytokines such as IL-17A, IL-1β, IL-6, IL-33, CCL-2, IFN-γ, and TNF-α [264, 266, 267]. Curcumin can also markedly upregulate CD3^-^CD19^+^CD1d^+^, CD3^-^CD19^+^CD25^+^, CD3^-^CD19^+^Foxp3^+^Breg cells level and significantly down-regulate CD3^-^CD19^+^PD-L1^+^, CD3^-^CD19^+^tim-1^+^, CD3^-^CD19^+^CD27^+^Breg cells level in DSS-induced colitis mice [264]. In addition, curcumin observably inhibited the key molecules in the TLR/MyD88 pathway. These findings suggest that curcumin can regulate the differentiation and function of Breg cell to alleviate DSS-induced colitis mice, which may be realized by inhibiting TLR/MyD88 pathway. Additionally, curcumin has anti-inflammatory and immunomodulatory effects on UC, which is related to its ability to regulate the function of DCs and Treg/Th cells [265, 266, 268].

As a result, it is considered a prominent area of research for potential therapeutic drugs targeting UC [244, 269]. Although these studies have shown the great potential of curcumin in the treatment of UC, more rigorous and in-depth clinical controlled trials are still needed for verification due to the non-standardized dosage forms, dosages, treatment timing, and administration routes in the clinical trials [259, 260].

#### Cool herbs

In traditional medical theory, cool herbs could clear heat and generate fluids. Pueraria, also known as Lobed Kudzuvine Root, is the dried root of the *Pueraria lobata* (Willd.) Ohwi from Leguminosae. In traditional medicine, pueraria has often been used for its ability to treat digestive diseases like diarrhea and UC. And puerarin is the most bioactive and abundant isoflavone compound in pueraria root, and the content of puerarin accounted for at least 2.4% of puerarin dried product [270] (Fig. 5). Puerarin has been used in the treatment of diseases such as cardiovascular diseases and neuroprotective treatments, due to its anti-inflammatory and antioxidation [271–273]. Similarly, the potential of puerarin in the treatment of UC has also been explored.

Studies have found that oral administration of puerarin (10 mg/kg and 50 mg/kg) could improve the general symptoms and disease activity of DSS-induced UC, possibly through down-regulating the levels of inflammatory cytokines like TNF-α, IL-1β and IL-6, transcription factor NF-κB, and other factors such as COX-2, PGE2, NO [250]. Secondly, the mechanism is to regulate the expression of the nuclear factor erythroid 2-related factor 2 (Nrf2) pathway and antioxidant-related enzymes such as, Nrf2, heme oxygenase (HO)-1 and NAD(P)H: quinone oxidoreductase 1 (NQO1), malondialdehyde (MAD), and other related factors [249, 250]. At the same time, it can enhance the the activities of antioxidant-related enzymes including CAT, glutathione (GSH), and SOD in a dose-dependent manner [250]. Peng et al. [251] found that puerarin (2, 4, and 8 μM) may improve LPS-induced inflammation injury in gastric epithelial cells GES-1, and decrease expression of NLR family pyrin domain containing 3 (NLRP3), ASC, cleaved caspase-1, IL-1β and IL-18 by activating AMPK/SIRT1 signaling pathway and inhibiting NLRP3 inflammasoma-mediated apoptosis. These results also confirmed the anti-inflammatory and anti-oxidative effects of puerarin in the digestive tract inflammation model. In addition, puerarin inhibited intestinal epithelial barrier dysfunction by increasing the expression of TJs (ZO-1, occludin and claudin-1) [249, 250].

In addition, pueraria was historically documented that could be used for the treatment of alcoholism in China. And now it is generally believed that alcohol is harmful to the human gut, leading to a substantial number of IBD patients voluntarily refraining from alcohol consumption [274]. Research studies have reported that the consumption of wine and beer might exacerbate endoscopic disease activity in patients with UC. Additionally, a higher intake of alcoholic beverages containing sulfites may elevate the risk of clinical relapse [275, 276]. DAI, colonic pathology and the reduction of colitic-mediated enzymatic and non-enzymatic antioxidants were significantly worse in 40% ethanol (2.5, 5 g/kg) -treated DSS-induced colitis mice than in DSS-alone group [277]. And Fan et al. [278] discovered that long-term alcohol consumption can lead to significant changes in the composition and structure of the microbiota in the colon. This is manifested by an increase in the phylum *Bacteroidetes* and eight specific genera, namely *Bacteroidales* S24-7, *Ruminococcaceae*, *Parabacteroides*, *Butyricimonas*, among others, as well as a decrease in the abundance of the *Lactobacillus* and *gauvreauii* genera. These alterations in the intestinal flora structure caused by alcohol consumption may play a crucial role in the development of intestinal injuries. Furthermore, the simultaneous administration of 25% ethanol (at a single dose of 2.9 g/kg) and a 12.5% body area burn in mice resulted in a decrease in the expression of TJs and mucins, specifically affecting the genes claudin-4, claudin-8 in both ileal and colonic epithelial cells, as well as claudin-2 and mucin-3 in the intestinal tract [279]. Present pharmacological research suggests that puerarin has an inhibitory effect on alcoholism and alcohol preference. Tectoridin (25, 50 and 100 mg/kg), an isoflavone glycoside from the flower of pueraria, could significantly reduce the levels of serum alanine transaminase (ALT), aspartate aminotransferase (AST), and triglyceride (TG) by dose-manner, meanwhile reverse alcoholic steatosis in mice oral administrated with ethanol (5 g/kg), through reversing the decrease in the levels of medium-chain acyl-CoA dehydrogenase, acyl-CoA oxidase and cytochrome P450 4A in peroxisome proliferators-activated receptor α (PPAR-α) and its related target genes [280]. Additionally, puerarin was reported to directly benefit diabetes mellitus and a series of complications by decreasing blood glucose levels, improving insulin resistance, protecting islets, inhibiting inflammation, decreasing oxidative stress, and inhibiting Maillard reaction and advanced glycation end products (AGEs) formation [281].

#### Tonifying herbs

In traditional medical theory, these herbs are used to replenish and strengthen Qi and blood. *Panax ginseng* C. A. Mey., known as ginseng, is a perennial herb of the genus Panax in the Araceae family. In traditional medicine, ginseng is considered to have the functions of tonifying vital qi, replenishing pulse, strengthening spleen and lung, nourishing body fluid and blood, calming mind and enhancing intelligence [205]. Currently, ginsenoside-based health care products have gained popularity in numerous countries. Ginsenoside, a crucial compound found in ginseng, has garnered extensive attention and utilization due to its anti-inflammatory properties and various other effects [252–255, 282, 283]. The chemical structure of ginsenoside is shown in Fig. 5.

Ginsenoside Rd (Rd) at a dose of 20 mg/kg effectively reduced inflammation in DSS-induced mice with colitis, and Rd downregulated the levels of pro-inflammatory cytokines including TNF-α, IFN-γ, IL-6, and IL-17A, and IL-12/23 p40, in vivo intestinal and peritoneal macrophages and in vitro isolated intestinal and peritoneal macrophages [252]. Furthermore, a decrease in the nuclear expression of p jun N-terminal kinase (JNK), p-p38, pIκBα, and P65 was observed in western blot analysis. These findings suggest that Rd can downregulate cytokine secretion by macrophages, alleviate DSS-colitis in mice, and potentially through the involvement of NF-κB and p38 mitogen-activated protein kinase (p38MAPK) pathways.

Ginsenoside Rc (Rc) at a dosage of 20 mg/kg significantly ameliorated DSS-induced decreases in body weight, colon weight and length, and increased DAI in mice. And Rc demonstrated the capacity to significantly reduce levels of inflammatory markers including TNF-α, IL-6, IL-1β, and NF-κB, both in vitro and in vivo. Rc also maintained intestinal barrier function by increasing the expression of claudin-1, occludin, and ZO-1 [253]. The signaling pathway of Farnesoid X receptor (FXR) has the ability to inhibit NF-κB, thereby reducing the expression of inflammatory factors such as TNF-α. The Bile salt export pump (BSEP) and small heterodimer protein (SHP) are known targets of the FXR signaling pathway, and their transcriptional induction expression is regulated when the FXR signaling pathway is activated [284]. In this study, it was observed that the expression of BSEP, SHP, and FXR was upregulated. Additionally, both the molecular docking assay and the FXR-knockout assay demonstrated that Rc could activate the FXR signaling pathway, resulting in the inhibition of NF-κB and subsequently reducing inflammation [253].

Ginsenoside Rg1 (Rg1), a natural compound with limited bioavailability, has demonstrated its efficacy in reducing colonic injury and inflammation, as well as balancing the gut microbiota structure and regulating tryptophan metabolism in DSS-induced UC mice [255]. Subsequently, in another study, Long et al. [254] confirmed this finding. In colitis mice, Rg1 exhibited a decrease in the levels of pro-inflammatory cytokines, such as IL-6, IL-33, CCL-2, and TNF-α, along with an increase in the levels of anti-inflammatory cytokines IL-4 and IL-10. Furthermore, aberrant M1/M2 macrophage polarization is involved in the pathogenesis of UC. Rg1 significantly downregulated M1 macrophages and upregulated M2 macrophages to modulate the balance between macrophage M1/M2 polarization [254]. Moreover, it also improved colonic microbiota diversity and regulated specific genera such as *Lachnospiraceae*, *Staphylococcus*, *Bacteroides*, and *Ruminococcaceae*_UCG_014 in colitis mice [254].

In addition, ginsenoside Rh2 has been demonstrated to possess anti-inflammatory effects in experimental colitis by suppressing the STAT3/miR-214 signaling pathway [282]. Similarly, ginsenoside Rk2 may exert its anti-inflammatory effects by attenuating the extracellular signal-regulated kinase/mitogen-activated protein kinase pathway through upregulating SIRT1 in vitro [283].

### Microecologics and fecal microbiota transplantation

Microecologics, including probiotics, prebiotics, synbiotics, and FMT, represent promising approaches for managing UC. These therapies aim to restore or enhance the gut microbiota, which plays a crucial role in UC pathogenesis.

#### Probiotics

Probiotics are living microorganisms found in specific foods and supplements. They have the potential to alleviate symptoms in individuals with UC by stimulating the growth of beneficial gut bacteria and reducing inflammation, particularly during active UC and remission phases [285–289]. There have been studies showing the therapeutic benefit of probiotics either alone or in conjunction with standard-of-care therapy in UC [286, 290].

Studies have demonstrated the inhibitory effect of probiotics on TLR signaling pathways in vitro in intestinal epithelial cells and DCs [291–293]. While a strain of *Lactobacillus* exerts beneficial effects in experimental necrotizing enterocolitis through modulation of TLR4 and NF-κB [294], a different strain acts through TLR2 in a radiation injury model. In addition, production of defensins was also upregulated in vitro in response to probiotic treatment [292]. Another strain of probiotic, *Bifidobacterium breve*, was able to reduce susceptibility to colitis in Nod1 and Nod2 mice ^-/--/-^ [295]. *Akkermansia muciniphila* (*Akk*) is regarded as a promising probiotic that could protect against colitis via the regulation of the immune response [296]. live *Akk*, pasteurised *Akk* and *Akk*’s outer protein Amuc_1100 could upregulate aryl hydrocarbon receptor (AhR) targeted genes, including *CYP1A1*, *IL-10* and *IL-22*, suggesting that *Akk* could activate AhR signaling by regulating tryptophan metabolism, thereby attenuating colonic inflammation [296]. Additionally, Qian et al. [297] found Amuc_2109 (β-N-acetylhexosaminidase from *Akk*) improved DSS-induced colitis as evidenced by lowered DAI, reduced weight loss, and increased colon length. And Amuc_2109 inhibited the overexpression of inflammatory cytokines TNF-α, IL-1β, and IL-6, and the NLRP3 inflammasomes in DSS-induced colitis.

VSL#3, a multi-strain probiotic mixture, could also be efficacious in inducing remission among individuals with mild to moderate UC [286]. Turis et al. [298] conducted a double-blind randomized study to investigate standard pharmaceutical treatment plus VSL#3 supplementation versus placebo for 8 weeks and found a significant reduction in the DAI and rectal bleeding. Moltke et al. [299] conducted a Phase 2B, double-blind, RCT to evaluate the efficacy, safety and microbiome alterations associated with two dose levels of SER-287 (microbiome therapy containing a consortium of live bacterial spores), after pre-treatment with vancomycin, in active mild-to-moderate UC. And the results of SER-287and Placebo groups showed that almost the same outcomes in clinical remission and endoscopic improvement. Similarly, *Bifidobacterium* combine with *Lactobacillus* treatment versus placebo in quiescent UC were terminated early after the 48-week follow-up due to no significant difference was found between the two groups in relapse-free survival [300]. *Escherichia coli Nissle 1917* (*EcN*) showed poorer clinical outcomes with active UC even compared with the placebo group [153]. However, it is comparable to mesalazine in sustaining remission [301]. In addition, VSL#3 may be valuable in preventing exacerbations triggered by *EcN* in patients with quiescent UC [302].

There are contradictions in these studies, which may be related to the type of probiotics and the relationship between probiotics and intestinal flora, environmental factors, etc. ​Currently, it has been established that several probiotics, such as VSL#3, are beneficial in the treatment of UC and can alleviate the symptoms of UC by reducing the inflammatory response and immune response.

#### Prebiotics

Prebiotics are non-digestible carbohydrates that modulate the endogenous gut microbiota by selectively stimulating the growth of health-promoting bacteria already present in the colon [303]. And prebiotics, including oligosaccharides such as galactose oligosaccharides, barley foodstuff, fructooligosaccharides, and others, are metabolized by gut microorganisms to form SCFAs that influence the local microenvironment to preferentially favour growth of certain flora [158, 304].

Although prebiotics have shown promising effects in UC animal experiments [305–307], there is limited evidence for the use of prebiotics in UC. In the RCTs, prebiotics showed similar clinical efficacy to mesalamine, and synbiotics, combined with probiotics and prebiotics, could also significantly decrease the CAI, the levels of intestinal mucosal inflammatory cytokines and fecal calprotectin, but not DAI [308–312]. However, large, high-powered RCTs using prebiotics or synbiotics in UC are still lacking.

#### Fecal microbiota transplantation

FMT refers to a method to treat the underlying disease by restoring the damaged intestinal flora by giving donor feces. Intestinal microbiota is a key factor in UC, and FMT can improve the intestinal microbiota, which is considered to have the potential to treat UC [313].

In RCTs, FMT administered by retention enema have shown significant clinical remission and complete mucosal healing at week 7, and microscopic remission or response at week 8 in the absence of steroid remission [314–317]. However, these studies did not find consistent bacteria associated with disease response. There are more and more studies on FMT in the treatment of UC, and many meta-analyses have analyzed it. And a re-meta-analysis [318] involving 12 primary RCTs and 544 participants found a large advantage of FMT patients over control patients in inducing combined clinical and endoscopic remission, clinical remission, endoscopic remission, clinical response, and endoscopic response. And serious adverse events did not differ significantly between patients who received FMT and those who received placebo. However, it is not clear whether FMT is a transient improvement or an improvement of the underlying cause, and the long-term safety of FMT treatment must also be verified [319].

## Discussion and conclusion

This article provides an overview of the current molecular mechanisms underlying UC and explores the intervention treatments available. GWAS have identified numerous genetic loci linked to UC, shedding light on the genetic factors that contribute to disease risk and progression. Notably, variants within the HLA region and SNPs have been associated with increased susceptibility to UC. Familial studies further support the role of genetic predisposition, with first-degree relatives of UC patients showing a higher risk of developing the disease. Additionally, certain genetic markers, such as those in the HLA class II molecules and genes like *CARD9*, have been implicated in UC susceptibility, suggesting their involvement in gut immunity, autophagy, and microbial defense. And ethnic variations may play a role in genetic susceptibility. The presence of perinuclear ANCA has emerged as a potential biomarker for UC, correlating with disease activity and severity.

The potential immune mechanisms of UC include impacting the innate and adaptive immune response by the influence of the DCs function, dysregulation of the Treg/Th cells, promotion of pro-inflammatory factor secretion, reduction of anti-inflammatory factor secretion. And IL-22 has a dual role in UC, which can not only play a protective role by promoting the recovery of the intestinal barrier, but also may exacerbate the inflammatory response by upregulating neutrophil-active chemokines such as *Cxcl1*. Additionally, NF-κB and JAK/STAT signaling pathways play a central role in the pathological process of UC by regulating the expression of inflammatory factors. Abnormal JAK/STAT signaling can lead to defects in the differentiation and regulatory activities of T cells and increase the susceptibility to UC. Studies on IL-6 as a therapeutic target have also achieved good results, and JAK/STAT signaling pathway may be involved.

In UC, significant alterations in gut microbiota, known as microbial dysbiosis, are observed, characterized by reduced diversity in the phyla Firmicutes and Bacteroidetes, alongside an increase in Enterobacteriaceae. This imbalance is more pronounced in inflamed regions and distinct from the microbiota of healthy individuals. Additionally, UC is associated with a decrease in SCFAs, particularly butyrate, which are crucial for maintaining intestinal health. The reduction in SCFAs contributes to heightened inflammation and compromised gut barrier function. Furthermore, increased mucosal permeability in UC exposes the immune system to bacterial antigens, leading to abnormal immune responses that exacerbate inflammation and disrupt immune regulation.

The emergence of biological and small molecule drugs partially complements the shortcomings of conventional treatment. For instance, JAK inhibitors and IL-12/23 mAb have been developed based on the underlying mechanism. Many biological drugs, especially JAK1 inhibitor UPT, have been put into clinical use due to their good therapeutic efficacy and safety. Despite the development of new medications and therapeutic approaches, such as phosphodiesterase inhibitors and sphingosine receptor modulators, only approximately 40% of patients achieve clinical remission after one year, highlighting the need for new treatment modalities [320–322]. Evaluating novel, non-medication-based modalities, such as FMT, could offer valuable adjunctive therapies [322].

Microecologics and FMT are emerging as promising treatments for UC. Probiotics, including specific strains of *Lactobacillus* and *Akk*, have shown potential in reducing inflammation and promoting beneficial gut bacteria, though study results are inconsistent. Prebiotics, which promote the growth of beneficial bacteria by producing SCFAs, have shown promise in animal models, but evidence in humans is limited and further research is needed to confirm their efficacy. FMT has shown significant clinical and endoscopic remission in UC patients by restoring gut flora. However, its long-term safety and effectiveness require further investigation. Variability in donor microbiota and inconsistent results contribute to these challenges. While FMT can restore gut flora, its benefits may be temporary rather than sustained.

The medicinal and edible herbal medicines mentioned in this article have shown their efficacy in UC studies, and the mechanism may be through regulating inflammation and immune response, gut microbiota, oxidative stress response, and intestinal barrier function. Chili pepper or capsaicin is commonly believed to have adverse effects on the human intestine, particularly unsuitable for patients with gastrointestinal inflammation. This is due to the potential of chili pepper or capsaicin consumption to induce a burning sensation in the gastrointestinal tract. This pain and burning sensation primarily results from neural responses, specifically action potentials triggered by the binding of capsaicin to TRPV1. This differs from the pain and burning sensation resulting from chemical burns due to exposure to acids or bases. Low concentrations of capsaicin (3.2-640 μM) do not harm the gastric mucosa, and in fact, they may safeguard it against ethanol-induced damage through the stimulation of sensory nerve endings. Conversely, inflammatory conditions lead to an increase in gut expression of TRPV1 and TRPA1 [208]. The activation of TRPV1 in sensory neurons can result in the release of neuropeptides like calcitonin gene-related peptide and substance P. This release leads to vasodilation, plasma extravasation, leukocyte migration, and an increase in proinflammatory cytokines [208]. Capsaicin can modulate inflammation and alleviate pain in UC by acting on both receptors. Thus, selectively inhibiting TRPV1 and TRPA1 proteins to manage pain shows potential in colitis treatment.

Curcumin treatment for UC is promising, and some researchers have also modified curcumin to make its anti-colitis effect better. Liu et al. [323] found that oral administration of turmeric-derived nanoparticles with a specific population (TDNPs 2) in a mouse colitis model could improve gut inflammation and accelerate the resolution of colitis by regulating the expression of proinflammatory cytokines TNF-α, IL-6 and IL-1β as well as the antioxidant gene HO-1. The results obtained from NF-κB-RE-Luc transgenic mice suggest that TDNPs 2-mediated inactivation of the NF-κB pathway may be linked to the amelioration of colitis. This suggests that some monomeric or derived ingredients from food, such as TDNPs 2, may be a novel, natural colo-targeted therapeutic agent that may prevent colitis and promote wound repair in colitis, and is superior to artificial nanoparticles in terms of low toxicity and ease of large-scale production.

In some Asian countries, botanicals are often used to treat certain diseases in the form of a multi-drug formula. This method of treatment based on traditional medical theory is believed to be better for the treatment of diseases. Daikenchuto (DKT) comes from the Synopsis of Prescriptions of the Golden Chamber, which consists of ginger, prickly ash, and ginseng. DKT is usually used to help to alleviate gastrointestinal symptoms such as abdominal pain or diarrhea. And in Japan, DKT has been integrated into the modern medical system as a prescription drug and is widely used for gastrointestinal diseases such as postoperative intestinal obstruction [324].

Existing studies have found that DKT can also improve UC. Dried ginger and ginger polysaccharide show promise in treating UC by reducing inflammatory cytokines, regulating factors associated with the intestinal barrier, and modulating intestinal flora. And ginger contains capsaicin and curcumin, further indicating its potential as a drug for treating colitis. Additionally, ginseng, which contains a certain amount of ginsenoside, have also shown other advantages such as anti-inflammatory and regulation of macrophage M1/M2 polarization balance and intestinal flora homeostasis. A study [325] found that the combination of ginger-ginseng exhibited the ability to enhance the abundance of beneficial bacteria such as *Muribaculaceae Norank*, *Lachnospiraceae*, and *Akk* in the colon. Moreover, this herbal pair was found to reduce the levels of harmful bacteria including *Bacteroides*, *Parabacteroides* and *Desulfovibrio* in the colon. And metabolomics analysis showed that arachidonic acid metabolism, tryptophan metabolism, steroid biosynthesis and other metabolic pathways were related to the mechanism of action of ginger-ginseng herb pair. These findings suggest that regulating the gut microbiota-metabolite axis may be a potential target for the synergistic treatment of ginger-ginseng herb pair in UC. In addition, the essential oil extract of prickly ash has shown promising effects in immune regulation, inflammation regulation, modulation of intestinal flora, and maintenance of intestinal barrier function. This indicates its potential for the treatment of UC.

DKT has been reported to have a vasodilator effect and increase blood flow in the gut, possibly by activating TRPA1, which is expressed on intestinal epithelial cells. Subsequently, vascular peptide adrenomedullin (ADM) is released [326], which was identified as the most potent endogenous vasodilatory peptide found in the body [327]. Kaneko et al. [328] discovered that DKT exhibits the ability to mitigate weight loss and colon ulceration in models of TNBS/OXN-induced colitis. Additionally, [6]-shogaol and hydroxy α-sanshool were found to be associated with an increase in ADM (adenylate cyclase-activating polypeptide) production. In a mouse model of DSS-induced colitis [329], oral administration of DKT demonstrated improvements in colorectal stenosis, reduction in serum hemoglobin levels, and significantly increased expression of IL-10. And studies have indicated that DKT administration is associated with significantly prolonged survival. In addition, DKT was found to suppress the visceromotor response to colorectal distention in rats treated with DSS and TNBS. Furthermore, research has shown that DKT can reduce the eosinophil count in both models of UC [330]. The combinations of ginseng, ginger, and prickly ash exhibited anti-inflammatory effects and possessed potent vasodilation activity in experimental colitis. The combination of botanicals hold promise as potential drugs for treating UC, and may emerge as a future trend in disease treatment.

Herbs have a significant impact on the development and prognosis of patients with UC. These botanicals or their natural components have demonstrated anti-colitis potential in vitro and/or in vivo studies, and may serve as promising alternatives or supplements for clinical drug development. Current UC research focuses on understanding potential mechanisms, with treatments being developed based on these insights. Most herbal treatments are based on traditional medicine theories, with clinical effectiveness established first, followed by research into their pharmacological mechanisms. However, the complexity of herbal ingredients makes studying their efficacy and safety challenging. For the natural components with therapeutic effects, it is believed that future studies need to study deeply, and to improve the delivery route by combining nanotechnology and other methods to exert the best drug effect. In addition, the combination of multiple drugs also provides a new direction for future treatment ideas.

Additionally, certain protein post-translational modification pathways, including aberrant ubiquitination, phosphorylation, methylation, and glycolysis, are associated with dysregulated immune responses and inflammation in IBD [331]. For example, mesenchymal stem cells regulate the ubiquitination of key signaling molecules in inflammatory pathways via the ubiquitin-proteasome system, which is responsible for protein degradation [331]. Therefore, developing interventions targeting these protein post-translational modification pathways to modulate the immune response and inflammatory conditions in IBD is promising.

Overall, by integrating molecular insights with therapeutic advancements, we aim to provide a comprehensive understanding of UC pathogenesis and management, ultimately contributing to improved patient outcomes and quality of life.

## Supplementary Information

Supplementary Material 1.

## Data Availability

No data was used for the research described in the article.
