# Supplementary material for: Ulcerative colitis: molecular insights and intervention therapy
Source: Mol Biomed. 2024 Oct 10;5:42. doi: 10.1186/s43556-024-00207-w (PMC11464740; doi:10.1186/s43556-024-00207-w)
Supplement: Supplementary file 1 — Supplementary Material 1. [file 43556_2024_207_MOESM1_ESM.pdf]

Supplementary Material

Confirmation of publication and licensing rights are provided by BioRender for publishing figures in the manuscript.

A CC-BY-NC-ND license is provided by BioRender for publishing figures in the manuscript.

49 Spadina Ave. Suite 200
Toronto ON M5V 2J1 Canada
www.biorender.com

Confirmation of Publication and Licensing Rights

August 22nd, 2024
Science Suite Inc.

Subscription:	*Student Plan - Academic*
Agreement number:	*TM277PRA9Y*
Publication name:	*Molecular Biomedicine*
Citation to Use:	*Created with BioRender.com*

To whom this may concern,

This document is to confirm that Yuqing Liang has been granted a license to use the BioRender Content, including icons, templates, and other original artwork, appearing in the attached Completed Graphic pursuant to BioRender's Academic License Terms. This license permits BioRender Content to be sublicensed for use in publications (journals, textbooks, websites, etc.).

All rights and ownership of BioRender Content are reserved by BioRender. All Completed Graphics must be accompanied by the following citation: "Created with BioRender.com".

BioRender Content included in the Completed Graphic is not licensed for any commercial uses beyond use in a publication. For any commercial use of this figure, users may, if allowed, recreate it in BioRender under an Industry BioRender Plan.

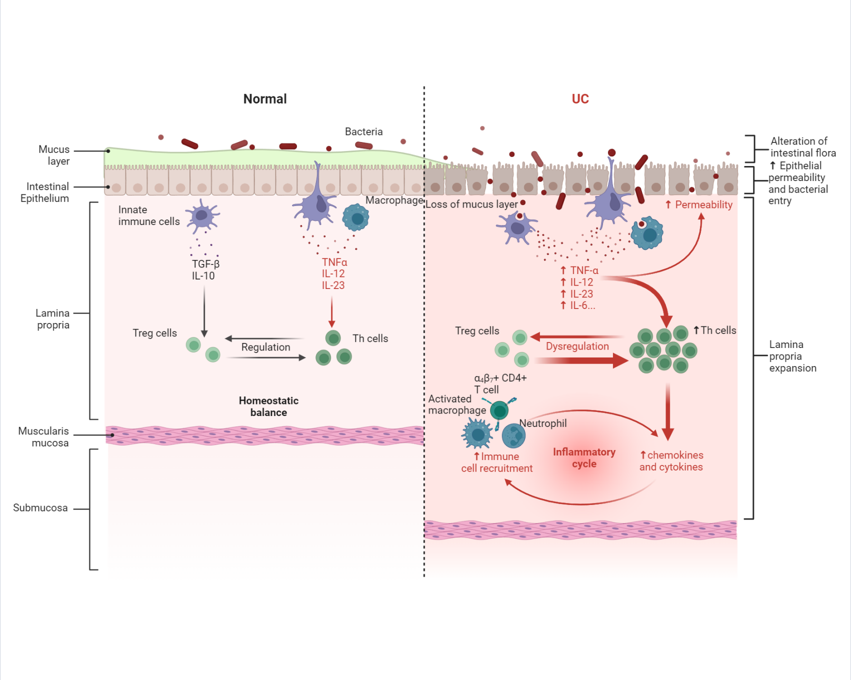

For any questions regarding this document, or other questions about publishing with BioRender refer to our BioRender Publication Guide, or contact BioRender Support at support@biorender.com.

49 Spadina Ave. Suite 200
Toronto ON M5V 2J1 Canada
www.biorender.com

Confirmation of Publication and Licensing Rights

August 22nd, 2024
Science Suite Inc.

Subscription:	*Student Plan - Academic*
Agreement number:	*YD277PH5MX*
Publication name:	*Molecular Biomedicine*
Citation to Use:	*Created with BioRender.com*

To whom this may concern,

This document is to confirm that Yuqing Liang has been granted a license to use the BioRender Content, including icons, templates, and other original artwork, appearing in the attached Completed Graphic pursuant to BioRender's Academic License Terms. This license permits BioRender Content to be sublicensed for use in publications (journals, textbooks, websites, etc.).

All rights and ownership of BioRender Content are reserved by BioRender. All Completed Graphics must be accompanied by the following citation: "Created with BioRender.com".

BioRender Content included in the Completed Graphic is not licensed for any commercial uses beyond use in a publication. For any commercial use of this figure, users may, if allowed, recreate it in BioRender under an Industry BioRender Plan.

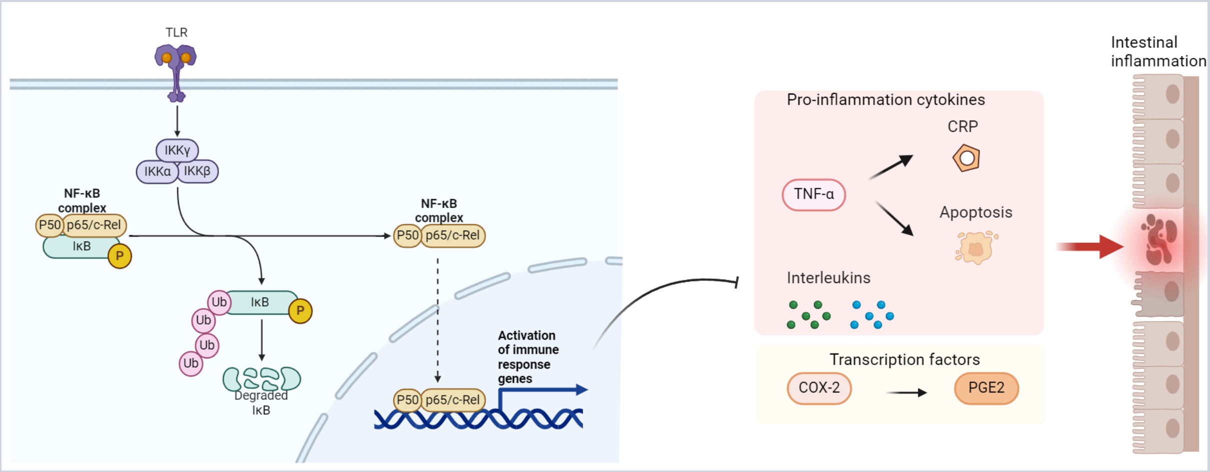

For any questions regarding this document, or other questions about publishing with BioRender refer to our BioRender Publication Guide, or contact BioRender Support at support@biorender.com.

49 Spadina Ave. Suite 200
Toronto ON M5V 2J1 Canada
www.biorender.com

Confirmation of Publication and Licensing Rights

August 22nd, 2024
Science Suite Inc.

Subscription:	*Student Plan - Academic*
Agreement number:	*XC277P2O9R*
Publication name:	*Molecular Biomedicine*
Citation to Use:	*Created with BioRender.com*

To whom this may concern,

This document is to confirm that Yuqing Liang has been granted a license to use the BioRender Content, including icons, templates, and other original artwork, appearing in the attached Completed Graphic pursuant to BioRender's Academic License Terms. This license permits BioRender Content to be sublicensed for use in publications (journals, textbooks, websites, etc.).

All rights and ownership of BioRender Content are reserved by BioRender. All Completed Graphics must be accompanied by the following citation: "Created with BioRender.com".

BioRender Content included in the Completed Graphic is not licensed for any commercial uses beyond use in a publication. For any commercial use of this figure, users may, if allowed, recreate it in BioRender under an Industry BioRender Plan.

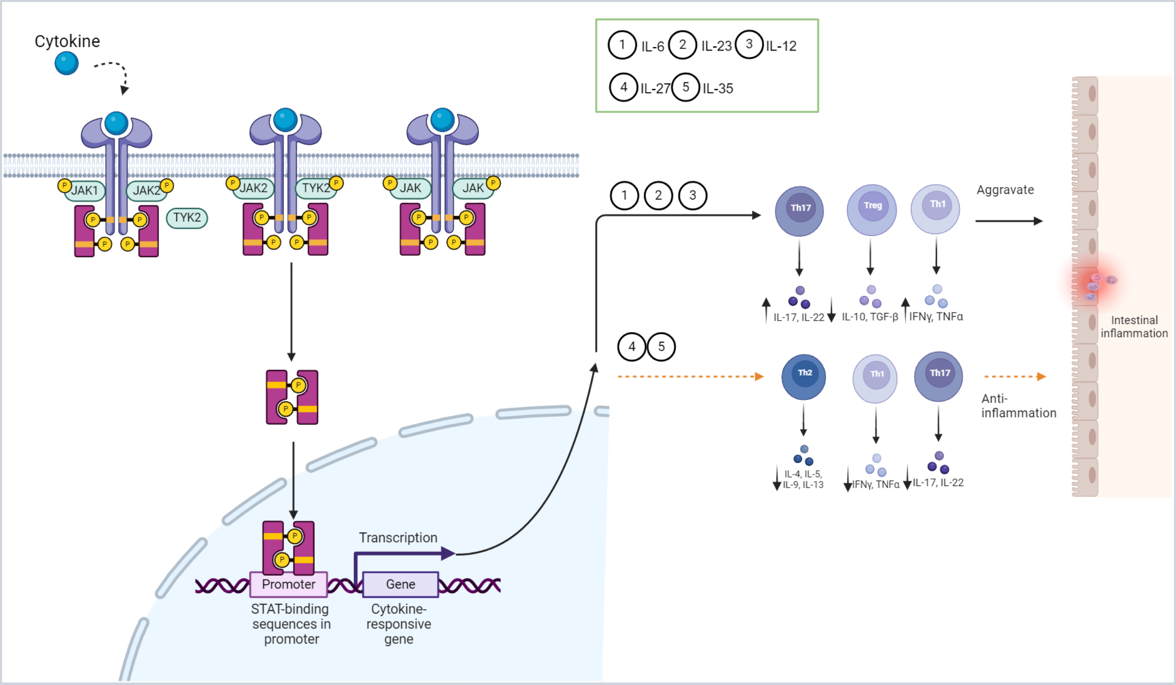

For any questions regarding this document, or other questions about publishing with BioRender refer to our BioRender Publication Guide, or contact BioRender Support at support@biorender.com.

49 Spadina Ave. Suite 200
Toronto ON M5V 2J1 Canada
www.biorender.com

Confirmation of Publication and Licensing Rights

August 23rd, 2024
Science Suite Inc.

Subscription:	*Student Plan - Academic*
Agreement number:	*DN277R77KX*
Publication name:	*Molecular Biomedicine*
Citation to Use:	*Created with BioRender.com*

To whom this may concern,

This document is to confirm that Yuqing Liang has been granted a license to use the BioRender Content, including icons, templates, and other original artwork, appearing in the attached Completed Graphic pursuant to BioRender's Academic License Terms. This license permits BioRender Content to be sublicensed for use in publications (journals, textbooks, websites, etc.).

All rights and ownership of BioRender Content are reserved by BioRender. All Completed Graphics must be accompanied by the following citation: "Created with BioRender.com".

BioRender Content included in the Completed Graphic is not licensed for any commercial uses beyond use in a publication. For any commercial use of this figure, users may, if allowed, recreate it in BioRender under an Industry BioRender Plan.

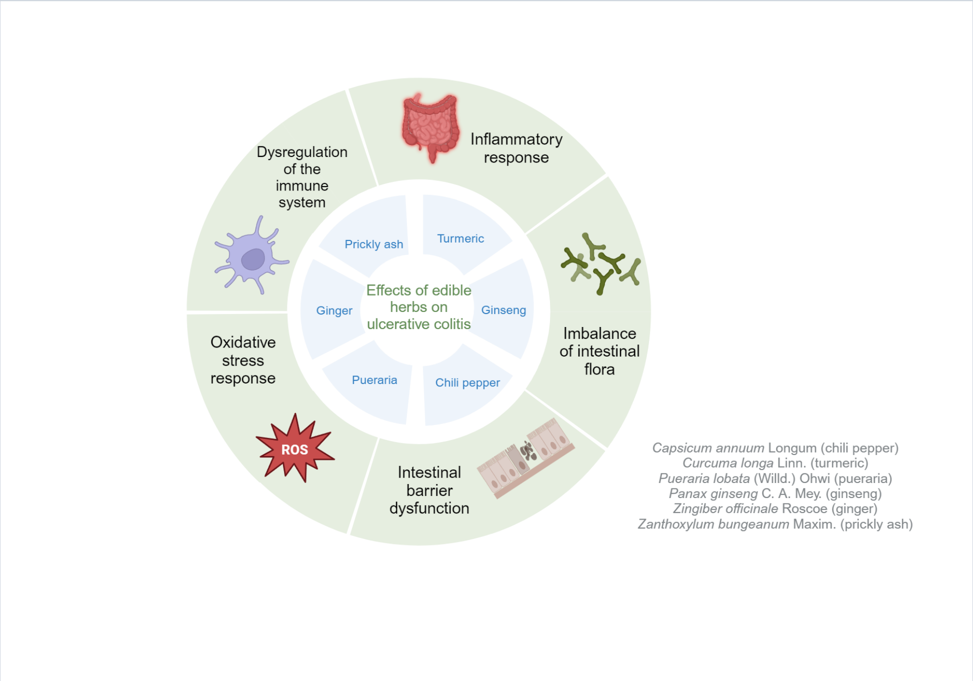

For any questions regarding this document, or other questions about publishing with BioRender refer to our BioRender Publication Guide, or contact BioRender Support at support@biorender.com.

49 Spadina Ave. Suite 200
Toronto ON M5V 2J1 Canada
www.biorender.com

Confirmation of Publication and Licensing Rights

August 23rd, 2024
Science Suite Inc.

Subscription:	*Student Plan - Academic*
Agreement number:	*UA277R7R71*
Publication name:	*Molecular Biomedicine*
Citation to Use:	*Created with BioRender.com*

To whom this may concern,

This document is to confirm that Yuqing Liang has been granted a license to use the BioRender Content, including icons, templates, and other original artwork, appearing in the attached Completed Graphic pursuant to BioRender's Academic License Terms. This license permits BioRender Content to be sublicensed for use in publications (journals, textbooks, websites, etc.).

All rights and ownership of BioRender Content are reserved by BioRender. All Completed Graphics must be accompanied by the following citation: "Created with BioRender.com".

BioRender Content included in the Completed Graphic is not licensed for any commercial uses beyond use in a publication. For any commercial use of this figure, users may, if allowed, recreate it in BioRender under an Industry BioRender Plan.

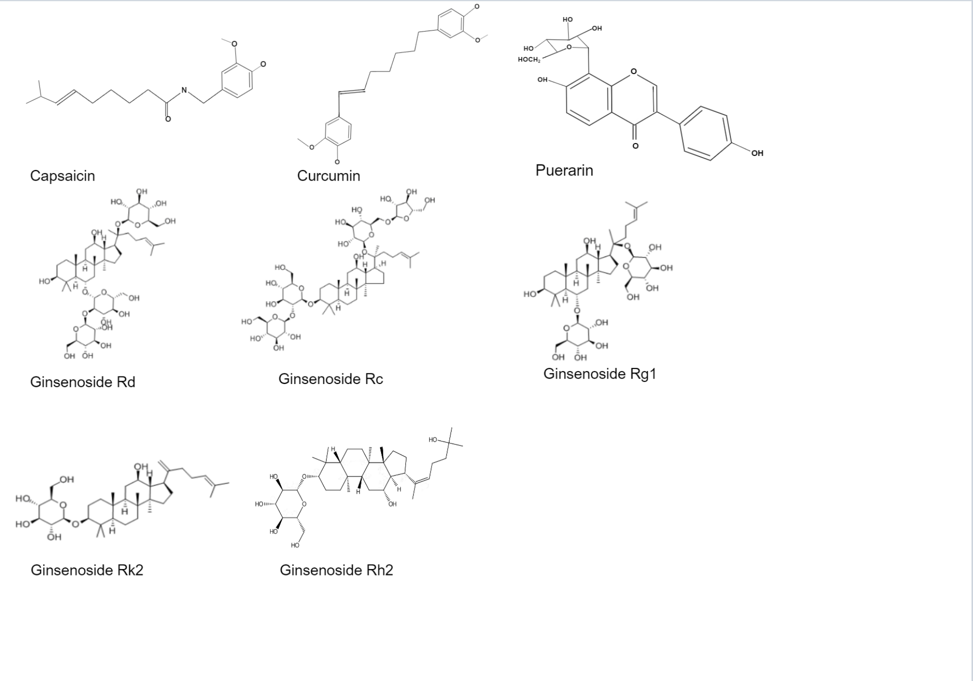

For any questions regarding this document, or other questions about publishing with BioRender refer to our BioRender Publication Guide, or contact BioRender Support at support@biorender.com.

BioRender Publication Licensing

The author **Yuqing Liang**, has a Premium subscription to BioRender (biorender.com). BioRender hereby grants **Molecular Biomedicine** the permission to publish the figure(s), and only figure(s):

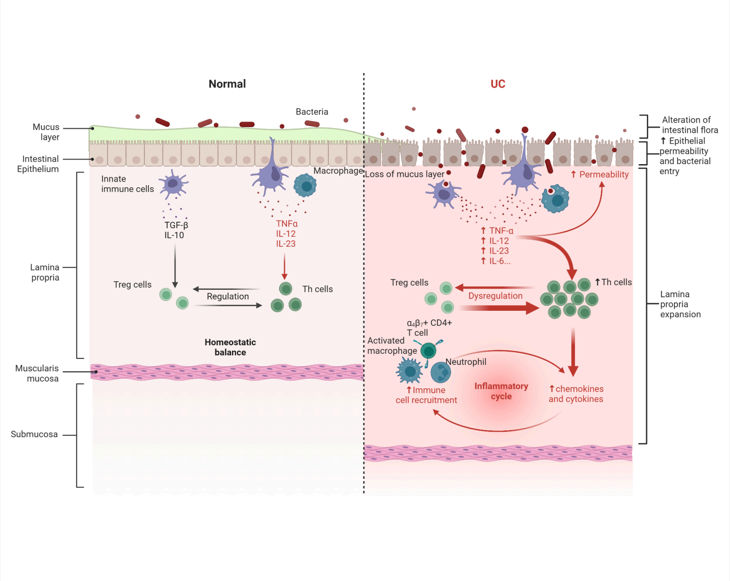

Figure 1. Potential immune response in UC

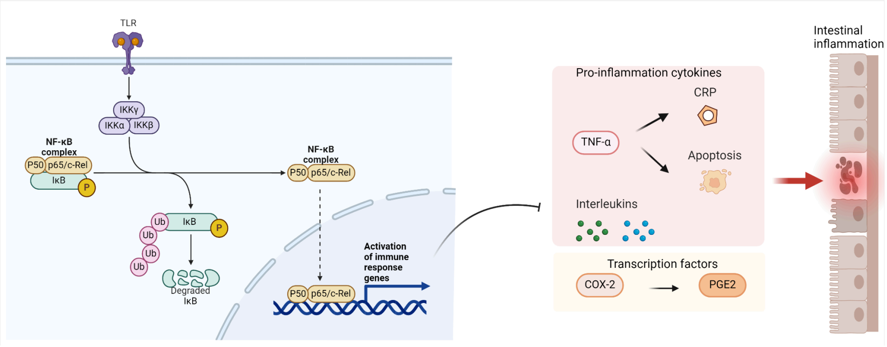

Figure 2. Activation of NF-κB signaling pathway increases the expression of pro-inflammatory genes to aggravate the inflammation of UC

bio
RENDER

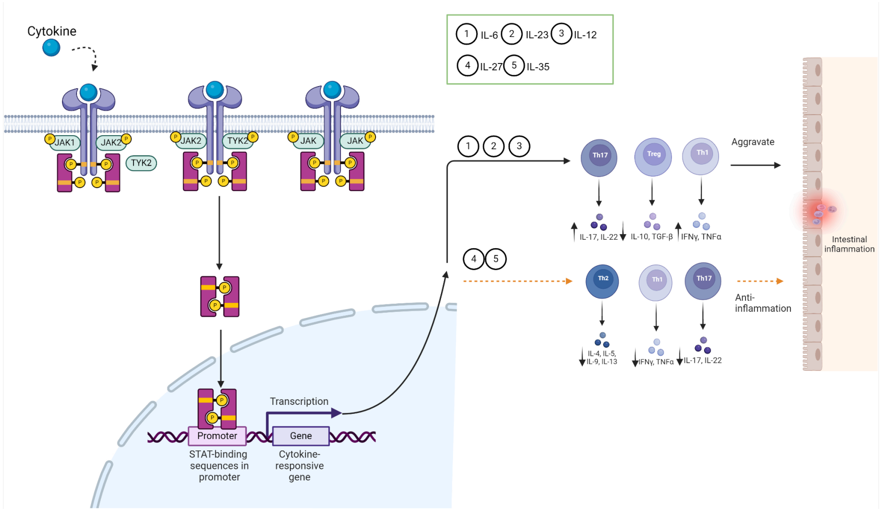

Figure 3. JAK/STAT signaling pathway can be activated or inhibited by inflammatory cytokines to regulate the inflammation of UC

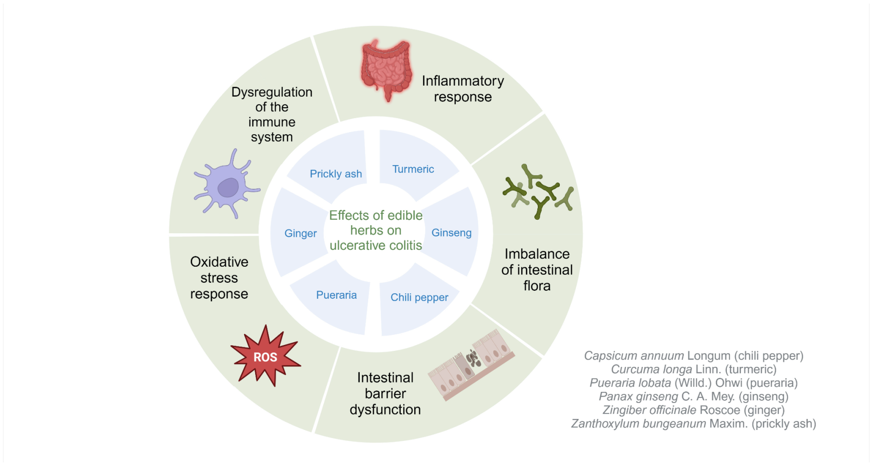

Figure 4. Effects of edible herbs on the UC

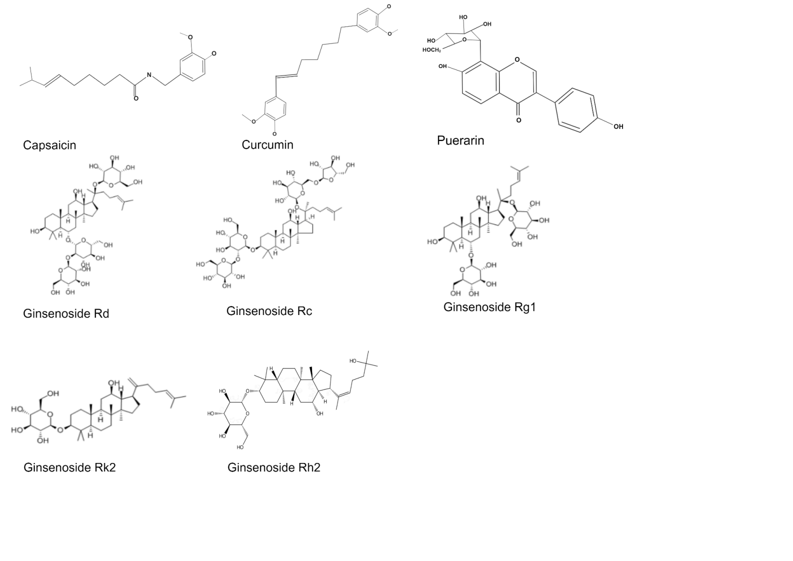

Figure 5. Chemical structures of capsaicin, curcumin, puerarin, and ginsenosides

In the manuscript **Ulcerative Colitis: Molecular Insights and Intervention Therapy** on a CC-BY-NC-ND license. This includes the permission to publish the figure(s) titled above in all formats including print and digital versions. The manuscript must include an acknowledgement of “Created with BioRender.com” on all versions of the publication to acknowledge that the figure(s) have been created using BioRender.

This permission is exclusively granted for the figure(s) titled above and does not supersede or modify in any way BioRender’s ownership, rights or license related to any of its intellectual property, as outlined in the BioRender terms and conditions located at https://biorender.com/terms/.

Mayet Awoke

Customer Experience Product Specialist

BioRender (Science Suite Inc.)

Mayet Awoke	Aug 27, 2024
Signature	Date
